# Genetic (Genomic) and Morphological Evidence Suggest That the Korean Endemic Fat Minnow (*Rhynchocypris kumgangensis*) and the Deogyu Population Represent Distinct Species

**DOI:** 10.1002/ece3.74253

**Published:** 2026-09-01

**Authors:** Soon Young Hwang, Ji Eun Jang, Ji Hyoun Kang, Hyuk Je Lee

**Affiliations:** ^1^ Molecular Ecology and Evolution Laboratory, Department of Biological Science Sangji University Wonju Republic of Korea; ^2^ National Park Research Institute Korea National Park Service Wonju Republic of Korea; ^3^ Korean Entomological Institute Korea University Seoul Republic of Korea

**Keywords:** allopatric speciation, climate‐sensitive biological indicator species (CBIS), Deogyu fat minnow, genetic/genomic adaptation, Korean endemic freshwater fish, phenotypic divergence

## Abstract

Geographically disconnected populations of freshwater fishes often show ecological and genetic divergence in response to opposing selection differentials in different habitat environments, sometimes leading to the formation of ecotypes and even speciation. Nevertheless, evidence for the speciation through allopatric processes in freshwater fish systems remains scarce. Kumkang fat minnow (*Rhynchocypris kumgangensis*), a Korean endemic coldwater fish, has recently been suggested to diverge into a separate species (*Rhynchocypris deogyuensis*). However, the level of their genetic and ecological divergence remains largely unknown. We analyzed population genetic structure of *R. kumgangensis* together with *R. deogyuensis* using mitochondrial DNA (mtDNA) and eight newly developed microsatellite markers. Genetic divergence at mitogenome level was further assessed between the presumed two species. Moreover, we examined morphology between two groups by analyzing morphometric traits and also conducted geometric morphometrics on body shape. We found distinct population structure between *R. kumgangensis* and *R. deogyuensis* at both mtDNA and microsatellites. One remnant population of *R. deogyuensis* harbored only a single haplotype and showed a very high level of inbreeding. Comparative analysis of the mitogenomes showed approximately 2.9% divergence between *R. kumgangensis* and *R. deogyuensis*, supporting the species‐level divergence. The analyses of both morphometric traits and geometric morphometrics indicated significant morphological divergence between the two species. The observed low values of length–weight relationship and condition factor for *R. deogyuensis* are likely to be attributed to effects of the elevated level of inbreeding and depleted genetic diversity. Overall, our combined genetic/genomic and morphological analyses suggest the considerable divergence between *R. kumgangensis* and *R. deogyuensis*, supporting the hypothesis that they are distinct species. The mechanisms underpinning how they speciate still need to be studied further in detail.

## Graphical abstract

Clear genetic and morphological divergence were revealed between the two Korean endemic 'putative' species *Rhynchocypris kumgangensis* and the Deogyu population (*R. deogyuensis)* using an integrated analysis of molecular markers, mitogenomes, and geometric and morphometrics on body shape. Pronounced population structure was also found within *R. kumgangensis* across geographic regions. These patterns suggest that speciation processes and restricted gene flow are acting concurrently and strongly among isolated populations inhabiting different river basins, providing important insights for freshwater fish conservation and management strategies in Korea.

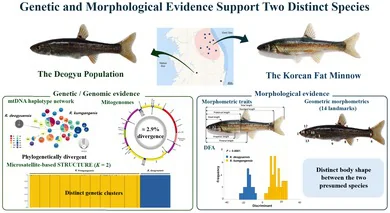

## 1 Introduction

The formation of new species through geographical isolation, that is, allopatric speciation has been widely regarded the most common mechanism for generating new species (Coyne 1992; Maderbacher et al. 2008; April et al. 2013; Seehausen and Wagner 2014; Alekseyev et al. 2023). Freshwater fish populations occurring in geographically disconnected river systems are restricted to migrate from one river/stream to another, unless human‐mediated translocation or overflow of rivers due to heavy rain and flooding. The geographical isolation may lead to divergent evolution and the formation of ecotypes among different river systems because of opposing selection differentials in different habitats and/or restricted gene flow (Ward et al. 1994; Kim et al. 2005; Choi and Lee 2025; Kim et al. 2026). The geological features of the Korean Peninsula, including a prominent mountain range lying to the east (i.e., the Baekdudaegan Mountain Range) and geographically isolated river systems, have restricted gene flow among freshwater fish populations and promoted morphological/ecological and genetic divergence among populations inhabiting different river basins (Lee et al. 2017; Kim et al. 2020; Jeon et al. 2020; Jeon et al. 2022). Such divergence associated with isolated river basins has been documented in several Korean freshwater fishes, including bitterlings *Tanakia latimarginata* (Kim et al. 2014), *Rhodeus pseudosericeus* (Arai et al. 2001), and *Tanakia signifer* (Choi and Lee 2025), a Korean spined loach *Cobitis choii* (Kim and Son 1984), and a Korean dark chub *Zacco koreanus* (Kim et al. 2020, 2026). There are 213 species of freshwater fishes on the Korean Peninsula, of which approximately 40% are indigenous to Korea, which represents extraordinarily high endemism (Choi 1973; Yoon et al. 2018). Endemic species are found to be geographically more restricted to small ranges. They generally have narrow ecological niches and are therefore more vulnerable to extinction than more widespread species (Gaston et al. 1997).

In recent decades, freshwater species diversity has been disappearing much faster than that of terrestrial and marine species (Harrison et al. 2018). The 2018 Living Planet Index (LPI) suggested that freshwater populations have diminished by an average of 83% during the past 50 years (WWF 2018). Biodiversity in freshwater fish habitats is greatly threatened by changes in environmental conditions, such as changes in flow and water quality (Ficke et al. 2007; Arthington et al. 2016; Reid et al. 2019). Freshwater fishes are poikilotherms that cannot regulate their physiological body temperature and therefore changes in environmental temperature cause shifts in the distribution and thereby diversity of freshwater fish communities (Ficke et al. 2007; Wood and McDonald 1997). In particular, for cold‐water fishes, shifts in species distribution caused by climate change‐driven habitat loss are directly linked to their survival (Schindler et al. 1990; Rahel et al. 1996; Stefan et al. 2001; Zheng et al. 2026).

**FIGURE 1:**
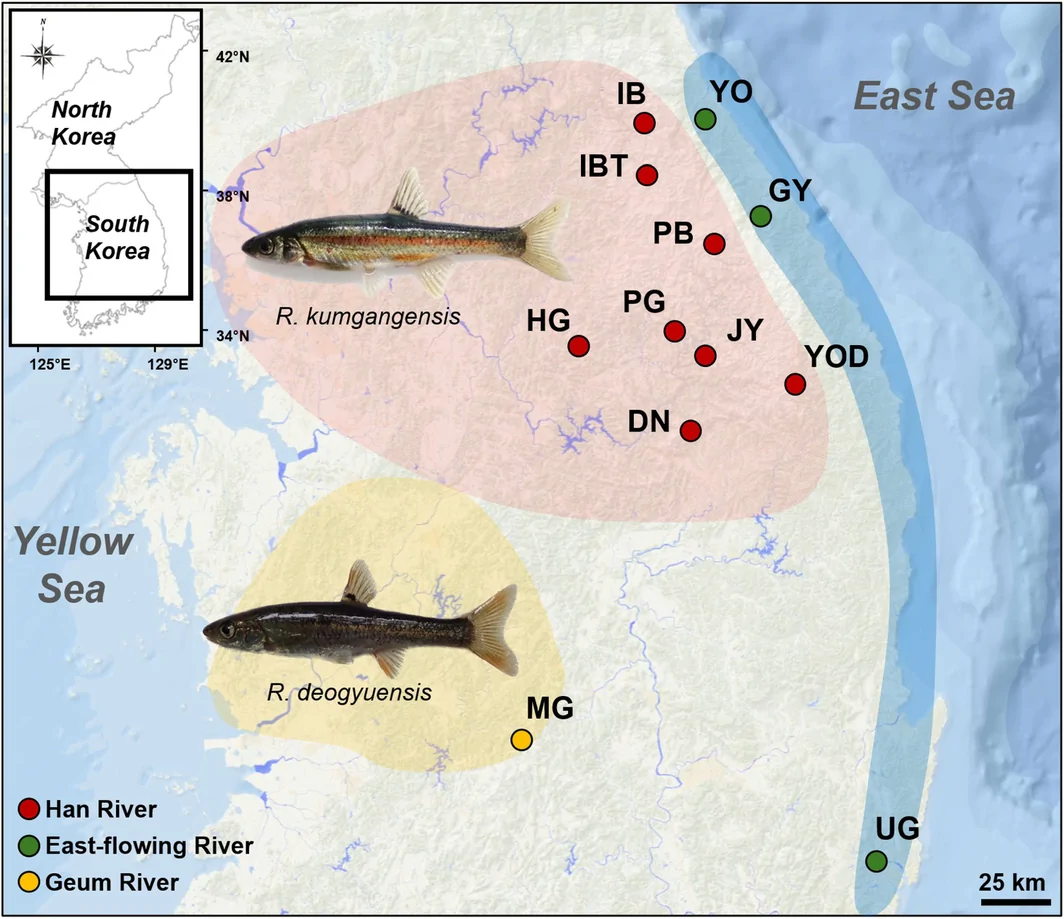
A map of sampling localities of *Rhynchocypris kumgangensis* and *R. deogyuensis* in South Korea. Detailed information on the sampling sites is given in Table S1. DN, Nam‐cheon; GY, Gangneung Yeongokcheon; HG, Hoengseong Gangrimcheon; IB, Inje Bukcheon; IBT, Inje Bangtaecheon; JY, Jeongseon Yongtancheon; MG, Muju Gucheondong Valley; PB, Pyeongchang Biancheon Stream; PG, Pyeongchang Gihwacheon; PH, Pyeongchang Heungjeongcheon; UG, Ulsan Guksucheon; YO, Yangyang Osaekcheon; YOD, Yeongwol Okdongcheon. Colored circles indicate sampling sites in different river basins.

The Kumkang fat minnow (*Rhynchocypris kumgangensis*; family Cyprinidae) is a cold‐water fish with a limited distribution at the uppermost parts of river and mountain streams located at high altitudes in the Daedong and Amnok Rivers in North Korea, and the Han and Geumgang Rivers in South Korea (Song 2000; Figure 1). Its occurrences in Guksucheon, Ulsan in the Nakdong River basin have recently been reported (Byeon 2016), although this species has not been naturally distributed there. As a cold‐water adapted fish, this species is known to be highly sensitive to ongoing climate change and has been designated as a Climate‐sensitive Biological Indicator Species (CBIS) since 2010 in Korea (NIBR 2010). Ecological analyses of *R. kumgangensis* have been conducted to understand reproductive characteristics (Song and Choi 1997; Song 2000), food source characteristics (Baek et al. 2002), habitat distribution (Byeon 2016; Jeon et al. 2019), effects of environmental changes on its ecological traits, and also effects of turbid water on fish community (Lee et al. 2008; Kim et al. 2013).

It has been suggested that the *R. kumgangensis* population inhabiting the Geum River is substantially genetically divergent from the population occurring in the Han River (Lee and Kim 2016). The geographically far isolated population distributed at the Gucheon‐dong Valley of Deogyusan National Park from the Geum River (Figure 1) has recently been classified as a separate species, *Rhynchocypris deogyuensis*, in 2017 based on analysis of genetic and morphological characteristics (Lee and Kim 2016; Lee and Sim 2017). However, given the low number of individuals included in the population genetic analysis for each population (*N* = 5–10) and the use of only a single mitochondrial DNA (mtDNA) marker, more comprehensive analyses are needed to test whether *R. kumgangensis* and *R. deogyuensis* show species‐level genetic and morphological divergence (Lee and Sim 2017). To date, their status as reproductively isolated, distinct species and the extent of their genetic/genomic and morphological/ecological divergence remain unresolved.

In this study, we conducted population genetic analyses of eleven populations of *R. kumgangensis* and the single currently known population of *R. deogyuensis*, using mtDNA cytochrome c oxidase I (COI) and control region (CR) sequences and eight newly developed microsatellite markers, to determine phylogeographic relationships and genetic structure within *R. kumgangensis* and between the two species. In addition, within‐population genetic diversity levels were analyzed and compared between the two species. We further assessed the level of genetic divergence between the two species at the whole mitogenome level. Moreover, we quantitatively analyzed seven morphometric traits and also conducted geometric morphometrics on their body shape to determine the ecological and morphological differences between *R. kumgangensis* and *R. deogyuensis*. The findings of this study provide interesting insights into the speciation process for Korean indigenous fishes and also highlight the urgent need for conservation and management efforts, given the extremely low genetic diversity, high inbreeding, and poor health conditions of *R. deogyuensis*.

## 2 Materials and Methods

### 2.1 Study Sites and Sample Collection

A total of 338 samples of *R. kumgangensis* (for mtDNA: *N* = 210; microsatellites: *N* = 219) and *R. deogyuensis* (mtDNA, microsatellites: *N* = 60) individuals were collected from 13 populations in South Korea between 2019 and 2023 using kick nets and cast nets (Figure 1, Table S1). The sampling sites for *R. kumgangensis* included the Han River basin (9 locations) and East–flowing River (3 locations) (Figure 1, Table S1). Its occurrences at the Ulsan Guksucheon (UG) in the Nakdong River have recently been reported (Byeon 2016), although this species had not been naturally distributed there. *Rhynchocypris deogyuensis* was sampled only at the Muju Gucheondong Valley (MG) in the Geum River basin because it only occurs there (Figure 1, Table S1). A majority of individuals were released immediately back to the collection sites after measuring weight and length and collecting fin clips. A total of sixty fish individuals from three populations (Pyeongchang Gihwacheon [PG; *N* = 20], Pyeongchang Heungjeongcheon [PH; *N* = 20], MG [*N* = 20]) were photographed live from a lateral view for the following geometric and morphometric analysis. Fin tissues were preserved in 99% ethanol and stored at 4°C until genetic analysis.

### 2.2 Genetic (and Genomic) Analyses

#### 2.2.1 Mitochondrial DNA Sequencing

Genomic DNA (gDNA) was extracted from the caudal fins using P&C Animal genomic DNA extraction kit (BIOSOLUTION, Suwon, Korea), according to its protocol guidelines. The extracted gDNA was checked using a ND‐1000 spectrophotometer (NanoDrop Technologies, USA) and kept of frozen at −20°C. MtDNA COI was amplified by polymerase chain reaction (PCR) using FishF1_n and FishR1_n primers (Ward et al. 2005). For mtDNA CR, we developed ourselves forward (RKCRF1; 5‐TTTTAACTCCCACCCCTGGC‐3) and reverse primers (RKCRR1; 5‐AGAACAGGATTCGGTGAGCG‐3) using the mitochondrial whole genome sequence information (AP011363, JQ675733) of *R. kumgangensis* registered in National Center for Biotechnology Information (NCBI). PCR amplification was carried out using a 2070 thermal cycler (Applied Biosystems, Waltham, USA) in a total reaction volume of 15 μL containing 10× Dream Taq Green buffer (Thermo Fisher Scientific, Waltham, USA), 2.0 mM dNTPs (Bio Basic Inc. Canada), 10 pmol each primer, 0.2 U of Taq polymerase (Thermo Fisher Scientific), and 4–30 ng/μL of DNA template. The following thermal cycling conditions were applied for COI and CR: initial denaturation phase at 95°C for 2–3 min, followed by 35 cycles of denaturation at 94°C for 30 s, annealing at 54°C for COI (57°C for CR) for 30 s, extension at 72°C for 1 min and final extension at 72°C for 10 min. PCR products were checked in 2% gels stained with Redsafe (iNtRON Biotechnology, Seongnam, Korea), purified enzymatically using Exonuclease I and Shrimp Alkaline Phosphatase (New England BioLabs, United States), and sequenced in a single forward direction using an ABI 3730xl Genetic Analyzer (Applied Biosystems) at the Humanizing Genomics Macrogen in Korea. The obtained DNA sequences were edited using GENEIOUS Prime 2022.1.1 (Biomatters Ltd., Auckland, New Zealand), and then manually verified.

#### 2.2.2 Microsatellites Development and Genotyping

We developed *R. kumgangensis* specific microsatellite markers using NGS (next generation sequencing)‐based sequencing from an MGI paired‐end library (2 × 150 bp paired‐end) using MGIEasy Universal DNA Library Prep Set (MGI, China) through the MGI MGISEQ‐2000 platform. The genome sequencing resulted in a total of 85,880,584 reads and 25,764,175,200 bp. We found 367,515 perfect microsatellite sequences, of which di‐nucleotides were the most abundant (299,352; 81.5%), tri‐nucleotides (34,441; 9.4%), and tetra‐nucleotides (28,203; 7.7%) were followed in this order. After testing levels of polymorphism and PCR yields at given 25 microsatellites, we finally chose eight loci (RKTCA13_F, RKAT20_F2, RKCT20_F, RKATCA9_F, RKTA20_F, RKAC18_F, RKAT20_F1, and RKTA_F1) for genotyping the samples. PCR was performed for 279 samples using eight primer sets, which were labeled with fluorescent dyes (FAM, HEX, and ATTO) on forward primers with the following conditions: an initial denaturation phase at 94°C for 5 min, followed by 35 cycles of denaturation at 94°C for 20 s, annealing at 45°C–54°C for 30 s, extension at 72°C for 12 min, and a final extension step conducted at 72°C for 10 min. The microsatellite fragment sizes were with the ROX 500 bp size standard (ABI) using GENEMAPPER software v5.0 (Applied Biosystem).

#### 2.2.3 Mitochondrial Genome (Mitogenome) Sequencing

Genomic DNA was isolated using a DNeasy Blood and Tissue Kit (Qiagen, Hilden, Germany), according to its protocol guidelines. Paired‐end sequencing for the mitogenome of three *R. deogyuensis* individuals was performed on the Miseq (Illumina Inc. San Diego, CA) platform. We obtained 1,805,675 raw read pairs with a length of 301 bp. These raw paired‐end read pairs were trimmed using Geneious Prime 2022.1.1 (Biomatters Ltd., Auckland, New Zealand) by error probability limit set to 0.05. The assembly and annotation of the mitogenome were also accomplished using the Geneious Prime. We compared the obtained mitogenomes of *R. deogyuensis* with the previously published mitogenome database of *R. kumgangensis* (accession nos. AP011363, JQ675733), which was suggested to be the most closely related species (Lee and Kim 2016). Using the mapped reads, de novo assembly was then performed with the default setting (assembler: Geneious, sensitivity: medium sensitivity/fast) of the Geneious. A circular mitochondrial genome map was drawn using OrganellarGenomeDRAW (OGDRAW) software v1.3.1 (Greiner et al. 2019).

### 2.3 Ecological (and Morphological) Analyses

#### 2.3.1 Length–Weight Relationship and Condition Factor

To assess and compare the length–weight relationship (LWR) and condition factor (*K*) between *R. kumgangensis* and *R. deogyuensis*, we collected biometric data (e.g., length, weight) from October 2022 to April 2023 before and after spawning periods of *R. kumgangensis* (PG and PH populations) and *R. deogyuensis* (MG population). The total length (TL) was measured using Image J (Abràmoff et al. 2004), and the weight was measured up to 0.01 g using an electronic scale (OHAUS, E02140, USA). The relationship between total length and weight followed the model of Anderson and Gutreuter (1983) [*W* = *aT*_*L*_^*b*^ (*W*: weight, *T*_*L*_: total length, *a*, *b*: parameter)]. The condition factor followed the model proposed by Anderson and Neumann (1996) [*K* = *W*/*T*_*L*_^3^ × 10^5^ (*W*: weight, *T*_*L*_: total length)]. The LWR and *K* values are known to represent fish's health conditions (e.g., fat content) (Fulton 1904).

#### 2.3.2 Measurements of Morphometric (Metric) Traits

To determine differences in the morphological characteristics between *R. kumgangensis* (PG and PH) and *R. deoyuensis* (MG) populations, seven traits were measured using photographs of collected individuals (PG: *N* = 30; PH: *N* = 30; MG: *N* = 30). External shape measurements were undertaken in Image J 1.49v. Head length (HL), body depth (BD), predorsal length (PDL), preanal length (PL), prepelvic length (PPL), and caudal peduncle depth (CPD) were calculated as ratios to standard length (SL), and eye diameter (ED) was calculated as a ratio to head length in Image J. Student's *t*‐tests were separately conducted for each of the seven traits to test whether there are significant differences in those metric traits between *R. kumgangensis* (PG and PH) and *R. deoyuensis* (MG).

#### 2.3.3 Analysis of Body Shape Using Geometric Morphometrics

**FIGURE 2:**
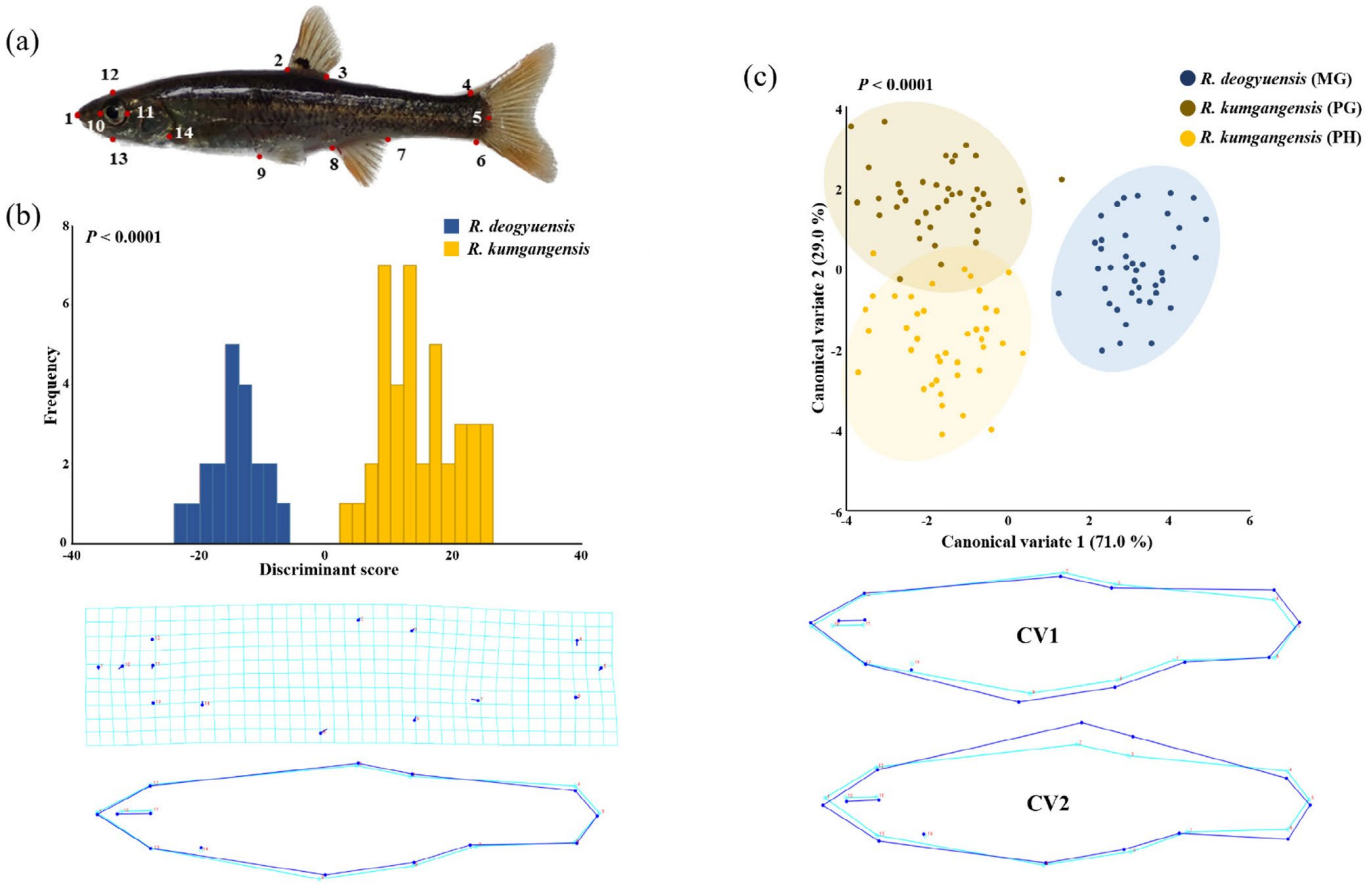
Geometric morphometric analysis of body shape variation between *Rhynchocypris kumgangensis* and *R. deogyuensis*. (a) Landmark configuration showing 14 anatomical landmarks used to define body shape. (b) Cross‐validated discriminant function analysis (DFA) distinguishing *R. kumgangensis* (yellow) and *R. deogyuensis* (blue). The deformation grid and wireframe diagram below the histogram illustrate shape changes associated with the discriminant function. (c) Canonical variate analysis (CVA) of three populations [*R. kumgangensis* (PG [*N* = 20], PH [*N* = 20]) and *R. deogyuensis* (MG; *N* = 20)]. Canonical variate 1 and canonical variate 2 explained 71.0% and 29.0% of the among‐population variation, respectively. The wireframe diagrams below the CVA plot illustrate shape changes along CV1 and CV2. In the deformation diagrams, light‐blue lines represent the reference mean shape, whereas dark‐blue lines represent the deformed shape along each axis.

Landmark‐based geometric morphometric analyses were performed to examine body shape differences among three populations of *R. kumgangensis* (PG and PH) and *R. deogyuensis* (MG) (Rohlf and Marcus 1993). Digital images were processed using TPSITIL v1.70 and fourteen landmarks (LMs) were positioned on digital photographs using TPSDIG v2.26 (Rohlf 2015) from each image (Figure 2a). Positions of the 14 LMs defining body associated with skeletal features were chosen since they had previously been used for the family Cyprinidae (Jalili et al. 2015). We utilized MORPHOJ v1.06d (Klingenberg 2011) to implement a generalized Procrustes superimposition approach, employing homologous landmarks (LMs). The Procrustes method for shape quantification effectively eliminated isometric size variation and minimized variation arising from differences in the positions and orientations of observed LMs (Rohlf 1999). Individual variation in body shape between/among populations was visualized using two types of analyses: (i) canonical variate analysis (CVA; for comparisons of three populations [*R. kumgangensis* (PG and PH) and *R. deogyuensis* (MG)]) and (ii) discriminant function analysis (DFA; for the comparison between *R. kumgangensis* and *R. deogyuensis*).

### 2.4 Data Analyses

#### 2.4.1 Genetic Diversity

For the concatenated mtDNA COI (590 bp) and CR (666 bp) sequences (1256 bp), the number of haplotypes (*N*_H_), haplotype diversity (*H*), and nucleotide diversity (*π*) were estimated for each population of *R. kumgangensis* and *R*. *deogyuensis* using ARLEQUIN v3.5 (Excoffier and Lischer 2010). To calculate haplotype richness (HR), the rarefaction method was applied using CONTRIB v1.02 (Petit et al. 1998), which corrected for unbalanced sample sizes across each population. To construct a haplotype network, HAPSTAR v0.7 (Teacher and Griffiths 2011) was used to infer the phylogenetic relationships among the haplotypes identified.

For eight microsatellite loci, the mean number of alleles per locus (*N*_A_), allelic richness (AR), which was corrected for unequal sample sizes, number of private alleles (PA), observed (*H*_O_) and expected (*H*_E_) heterozygosities, inbreeding coefficient (*F*_IS_), Hardy–Weinberg equilibrium (HWE) statistics were calculated using GENEPOP v4.0 (Rousset 2008) and FSTAT v2.9.3.2 (Goudet 2001). Contemporary effective population size (*N*_e_) was estimated for each population using NeEstimator v2.01 (Do et al. 2014). *N*_e_ was estimated using the linkage disequilibrium method with the lowest allele frequency threshold (*P*_crit_) of 0.02. The 95% confidence intervals (95% CI) were derived from jackknifing across all loci. Potential null alleles, large‐allele dropout, and scoring errors caused by stuttering were evaluated for each microsatellite locus and population using MICRO‐CHECKER v2.2.3 (Van Oosterhout et al. 2004). For this, frequencies of null alleles were estimated across the eight microsatellite loci and 12 populations.

#### 2.4.2 Genetic Differentiation and Population Structure

To examine the spatial genetic differentiation between *R. kumgangensis* (*N* = 11) and one *R. deogyuensis* (*N* = 1) populations, pairwise estimates of *F*_ST_ with haplotype (mtDNA) and allele (microsatellites) frequencies using ARLEQUIN and GENEPOP. The 95% significance levels for pairwise population comparisons were also corrected using the Bonferroni correction. The population genetic structure was further investigated through the application of a Bayesian model‐based clustering algorithm, implemented in STRUCTURE v2, assuming a model that considers admixed ancestry within each population and incorporates correlated allele frequencies. STRUCTURE calculates a likelihood score when the data are forced into a given number of genetic clusters (*K*) = 1–12. We applied 10 iterations, with 100,000 burn‐in steps followed by 1000,000 Markov Chain Monte Carlo (MCMC) generations. The most likely number of genetic clusters (*K* value) was determined by using the delta *K* (Δ*K*) method implemented in the web‐based tool STRUCTURE HARVERSTER website (http://taylor0.biology.ucla.edu/structureHarvester/), based on the rate of change in the log probability of data between successive *K* values (Earl and VonHoldt 2012). Moreover, we estimated patterns of genetic differentiation derived from genetic distance values among populations with a principal coordinate analysis (PCoA) using GenAlEx v6.51 (Peakall and Smouse 2006).

#### 2.4.3 Phylogenetic Analyses and Divergence Time Estimation

To determine phylogenetic relationships across 12 populations of *R. kumgangensis* and *R. deogyuensis*, concatenated sequences of COI and CR (1256 bp) was aligned with the outgroup sequence, resulting in a total alignment length of 1357 bp. The outgroup species included *Rhynchocypris oxycephalus* and *Phoxinus phoxinus* (Accession Nos.: MW057563.1, NC_020358.1). Statistical support was estimated by 1000 bootstrap replicates. Phylogenetic trees were separately constructed with neighbor joining (NJ), maximum likelihood (ML) and maximum parsimony (MP) methods using as implemented in Mega X (Kumar et al. 2018). Phylogenetic analysis was also conducted with a neighbor joining (NJ) approach, based on microsatellite allelic variation. D_A_ distances among the 12 samples were calculated using POPTREE2 (Takezaki et al. 2010). At the mitogenome level, genetic distance was estimated using Geneious Prime between *R. kumgangensis* and *R. deogyuensis* to test whether they showed the species‐level divergence in DNA sequences.

Bayesian divergence time estimation was conducted using BEAST v2.4.8 (Drummond et al. 2012). The dataset included 11 haplotypes of *R. kumgangensis*, one haplotype of *R. deogyuensis*, and two outgroup taxa, with two mitochondrial gene partitions (COI and CR). A single, linked tree topology was assumed for both gene partitions, while independent substitution models (GTR + Γ + I) and separate strict molecular clocks were adopted to each locus. Divergence time calibration was based on previously reported substitution rates for freshwater fishes: 1.5% per million years (0.015 substitutions/site/MYA) for COI (Adeoba et al. 2018) and 0.76% per million years (0.0076 substitutions/site/MYA) for CR (Wang et al. 2022). The Markov Chain Monte Carlo (MCMC) was run for 10 million generations, sampling every 1000 generations, with the first 10% discarded as burn‐in. Convergence and parameter mixing were assessed in Tracer v1.5, ensuring adequate effective sample size (ESS) values (> 200). The maximum clade credibility (MCC) tree with mean node heights was summarized using TreeAnnotator (part of the BEAST package) and visualized in FigTree v1.4.4.

## 3 Results

### 3.1 Genetic Diversity

**FIGURE 3:**
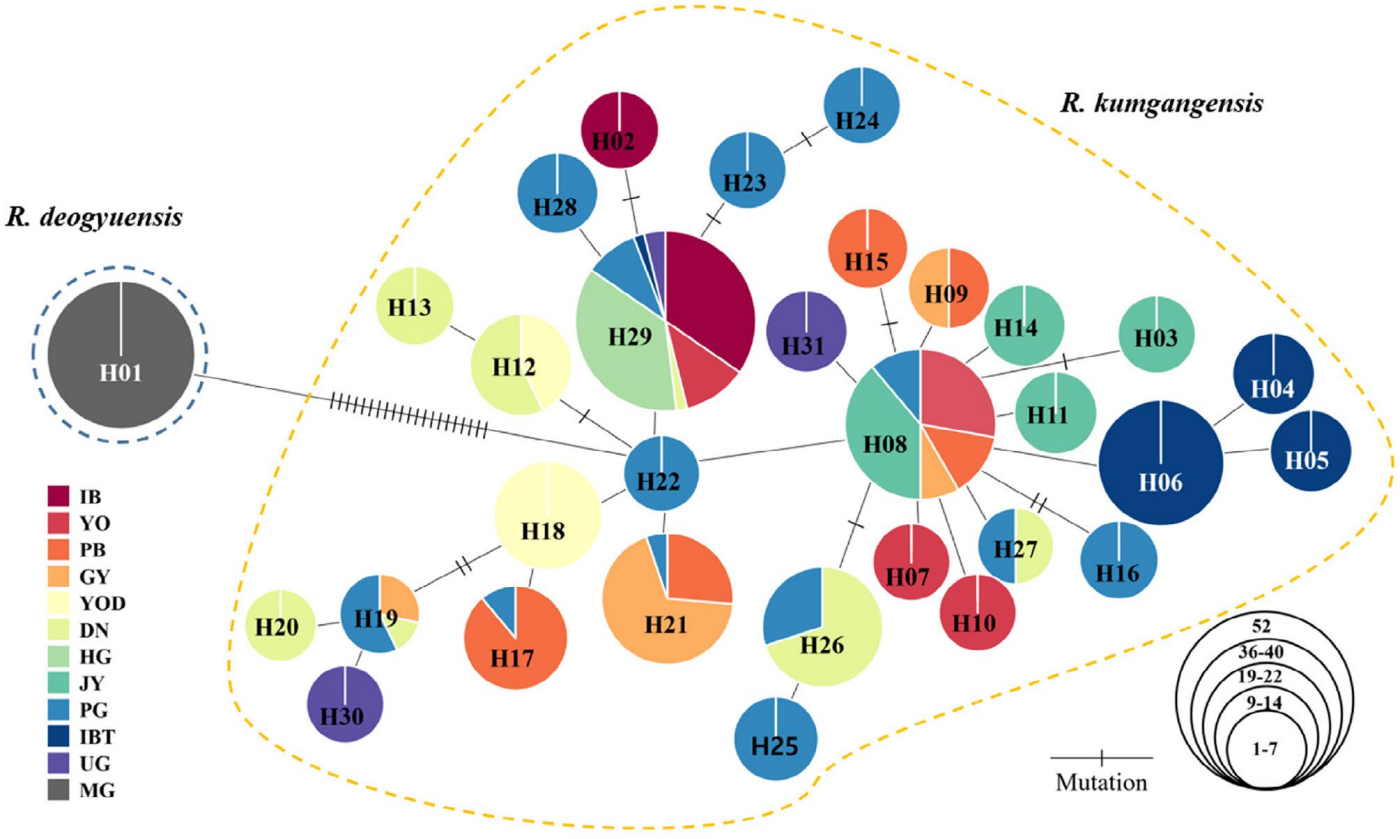
Haplotype networks of *Rhynchocypris kumgangensis* and *R. deogyuensis* based on mtDNA COI gene and mtDNA control region (1256 bp). A total of 31 haplotypes were detected in *R. kumgangensis* (30 haplotypes) and *R. deogyuensis* (only one haplotype; H01). The *R. deogyuensis* haplotype H01 diverged with 21 mutation steps from the closest *R. kumgangensis* haplotype H22. The size of the circle is proportional to the number of individuals that belong to the respective haplotypes.

A total of 30 mtDNA haplotypes were identified in 210 individuals from 11 populations of *R. kumgangensis*, whereas only one private haplotype was identified in 60 individuals from a single population of *R. deogyuensis* (Table 1, Figure 3). Genetic diversity indices, including *N*_H_, HR, H, π, and PH within the 12 populations of *R. kumgangensis* and *R. deogyuensis* showed mean *N*_H_: *R. kumgangensis*; 4.545 ± 3.2 and *R. deogyuensis*; 1.000, HR: 3.167 ± 2.7 and 0.000, H: 0.507 ± 0.3 and 0.000, π: 0.001 ± 0.0 and 0.000, PH: 1.909 ± 1.7 and 1.000, respectively (Table 1). Within *R. kumgangensis*, the PG population showed the highest HR and PH values (HR = 9.421; PH = 5), whereas the HG population showed the lowest diversities (HR = 0.000; PH = 0).

**TABLE 1:** Summary of genetic diversity in 12 populations of *Rhynchocypris kumgangensis* and *R. deogyuensis* in South Korea at both mtDNA COI and control region and microsatellite loci.

	Pop	Mt DNA						Microsatellites								
		*N*	*N*_H_	HR	PH	*h*	*π*	*N*	*N*_A_	*N*_e_	AR	PA	*H*_E_	*H*_O_	*F*_IS_	H‐W tests (*p*)
*R. kumgangensis*	IB	19	2	0.47	1	0.105	0.000	20	6.000	552.625 (4.41–∞)	5.779	7	0.708	0.608	0.146	*
	PB	20	5	3.800	1	0.747	0.002	20	8.250	∞ (94.8–∞)	7.941	4	0.764	0.515	0.334	*
	DN	20	7	5.500	2	0.742	0.003	20	6.250	∞ (52.6–∞)	6.302	3	0.734	0.585	0.207	*
	YOD	19	2	1.000	1	0.456	0.001	20	6.125	97.925 (27.7–∞)	5.868	1	0.660	0.550	0.170	*
	HG	19	1	0.000	0	0.000	0.000	20	4.750	174.725 (22.2–∞)	4.533	3	0.532	0.433	0.187	*
	JY	19	4	2.895	2	0.450	0.000	20	4.625	∞ (40.4–∞)	4.434	2	0.569	0.598	−0.052	NS
	PG	26	13	9.421	5	0.920	0.003	28	10.750	1651.575 (129.9–∞)	9.524	7	0.824	0.639	0.228	*
	IBT	25	4	2.160	2	0.230	0.000	25	6.625	∞ (99.2–∞)	6.022	5	0.654	0.474	0.276	*
	YO	18	4	3.000	0	0.608	0.001	20	5.875	39.25 (23.8–118.0)	5.733	1	0.711	0.563	0.214	*
	GY	19	4	2.947	0	0.521	0.001	20	5.500	41 (25.5–155.6)	5.310	2	0.709	0.583	0.184	*
	UG	6	4	—	2	0.800	0.002	6	6.125	∞ (34.7–∞)	—	9	0.826	0.667	0.208	*
Total	210	4.545 (mean)	3.167 (mean)	1.909 (mean)	0.507 (mean)	0.001 (mean)	219	6.443 (mean)	—	6.118 (mean)	—	0.699 (mean)	0.565 (mean)	—	—	
*R. deogyuensis*	MG	60	1	0.000	1	0.000	0.000	60	1.875	30.1 (3.2–∞)	1.705	9	0.146	0.110	0.248	*

Abbreviations: *π*, nucleotide diversity; AR, allelic richness; *F*_IS_, inbreeding coefficient; *h*, haplotype diversity; *H*_E_, expected heterozygosity; *H*_O_, observed heterozygosity; HR, haplotype richness; H‐W tests (*p*), *p* values for multi‐locus test for Hardy Weinberg equilibrium (HWE); *N*, sample sizes; *N*_A_, observed mean number of alleles across eight loci; *N*_e_, contemporary effective population size; *N*_H_, number of haplotypes; NS, not significant; PA, number of private alleles; PH, number of private haplotypes.

* *p* < 0.001 after a Bonferroni correction applied.

In the haplotype network analysis using COI and CR, 31 haplotypes were observed for the pooled dataset of *R. kumgangensis* and *R. deogyuensis*. The haplotype network analysis showed that *R. kumgangensis* and *R. deogyuensis* populations were clearly genetically diverged (Figure 3). All 60 individuals in *R. deogyuensis* displayed a single haplotype (H01) with 21 mutation steps from the closest haplotype (H22), and no haplotypes were shared between the two species. H29 and H08 are common haplotypes shared by seven and five populations of *R. kumgangensis*, and the majority represented a singleton haplotype (71.0%) (Figure 3).

Microsatellite diversity indices (*N*_A_, *H*_E_, *H*_O_, AR and HWE *p*‐values) and *N*_e_ were estimated for the *R. kumgangensis* and one *R. deogyuensis* populations and are summarized in Table 1. The estimates of null allele frequencies ranged from −0.127 (CT20; MG) to 0.360 (AT20; PB) (overall mean = 0.063), indicating a very low probability of null alleles present in our dataset. The mean values of *N*_A_, *H*_E_, *H*_O_ and AR within *R. kumgangensis* and *R. deogyuensis* were *N*_A_: 6.443 ± 1.6 and 1.875, *H*_E_: 0.699 ± 0.1 and 0.146, *H*_O_: 0.565 ± 0.1 and 0.110, AR: 6.118 ± 1.5 and 1.705 (Table 1). Similar to the mitochondrial diversity, the PG population showed the highest AR value (AR = 9.524), whereas the JY populations showed the lowest diversities (AR = 4.434). Interestingly, the UG population harbored the highest number of PAs, albeit its sample size analyzed is the smallest. An extraordinarily low level of microsatellite diversity in *R. deogyuensis* population resulted in a significantly high level of *F*_IS_ (*F*_IS_ = 0.25), although not the highest value (Table 1). The *N*_e_ estimate for *R*. *deogyuensis* was a median of 30.1, whereas *N*_e_ estimates for *R. kumgangensis* were generally higher and often unbounded, with a median of 426.2 when populations with infinite estimates were excluded (Table 1).

### 3.2 Genetic Differentiation and Population Structure

Pairwise *F*_ST_ values between populations at both mtDNA and microsatellite markers showed very high levels of genetic differentiation (Table 2). The pairwise estimates of *F*_ST_ between the 12 populations ranged from −0.058 to 0.923 (within *R. kumgangensis*), 0.956 to 1.000 (*R. kumgangensis* vs. *R. deogyuensis*) at mtDNA and 0.032 to 0.327 (within *R. kumgangensis*), 0.536 to 0.711 (*R. kumgangensis* vs. *R. deogyuensis*) at microsatellites. The observed level of genetic divergence may suggest at least the state of “strong, but reversible reproductive isolation” along the speciation continuum (Hendry et al. 2009).

**TABLE 2:** Pairwise genetic differentiation (*F*_ST_) between 11 populations of *R. kumgangensis* and one population of *R. deogyuensis*, based on mitochondrial DNA COI and CR (below diagonal) and eight microsatellite loci genotyped (above diagonal).

Population	IB	YO	PB	GY	YOD	DN	HG	JY	PG	IBT	UG	MG
IB		**0.125**	**0.148**	**0.206**	**0.196**	**0.186**	**0.242**	**0.270**	**0.110**	**0.200**	**0.114**	**0.634**
YO	**0.562**		**0.080**	**0.156**	**0.200**	**0.142**	**0.204**	**0.293**	**0.048**	**0.183**	**0.111**	**0.607**
PB	**0.543**	**0.233**		**0.067**	**0.180**	**0.114**	**0.202**	**0.257**	**0.032**	**0.142**	**0.078**	**0.587**
GY	**0.612**	**0.346**	**0.168**		**0.178**	**0.166**	**0.283**	**0.298**	**0.099**	**0.179**	**0.122**	**0.640**
YOD	**0.674**	**0.478**	**0.222**	**0.395**		**0.171**	**0.296**	**0.327**	**0.136**	**0.146**	**0.107**	**0.682**
DN	**0.325**	**0.180**	**0.237**	**0.292**	**0.234**		**0.198**	**0.229**	**0.058**	**0.134**	**0.068**	**0.617**
HG	0.000	**0.605**	**0.565**	**0.640**	**0.705**	**0.337**		**0.303**	**0.124**	**0.243**	**0.227**	**0.696**
JY	**0.833**	**0.197**	**0.354**	**0.491**	**0.612**	**0.300**	**0.871**		**0.178**	**0.271**	**0.233**	**0.695**
PG	**0.199**	0.061	**0.133**	**0.185**	**0.236**	0.077	**0.206**	**0.235**		**0.125**	**0.034**	**0.536**
IBT	**0.896**	**0.599**	**0.579**	**0.671**	**0.752**	**0.511**	**0.923**	**0.660**	**0.437**		**0.123**	**0.654**
UG	0.267	0.120	0.205	0.281	**0.359**	0.070	**0.321**	0.460	−0.058	**0.675**		**0.711**
MG	**0.998**	0.**989**	**0.977**	**0.983**	**0.986**	**0.963**	**1.000**	**0.994**	**0.956**	**0.995**	**0.990**	

*Note:* Population abbreviations indicated as in Figure 1. Significant pairwise *F*_ST_ and *p* values are bolded (*p* < 0.05) after the Bonferroni correction.

**FIGURE 4:**
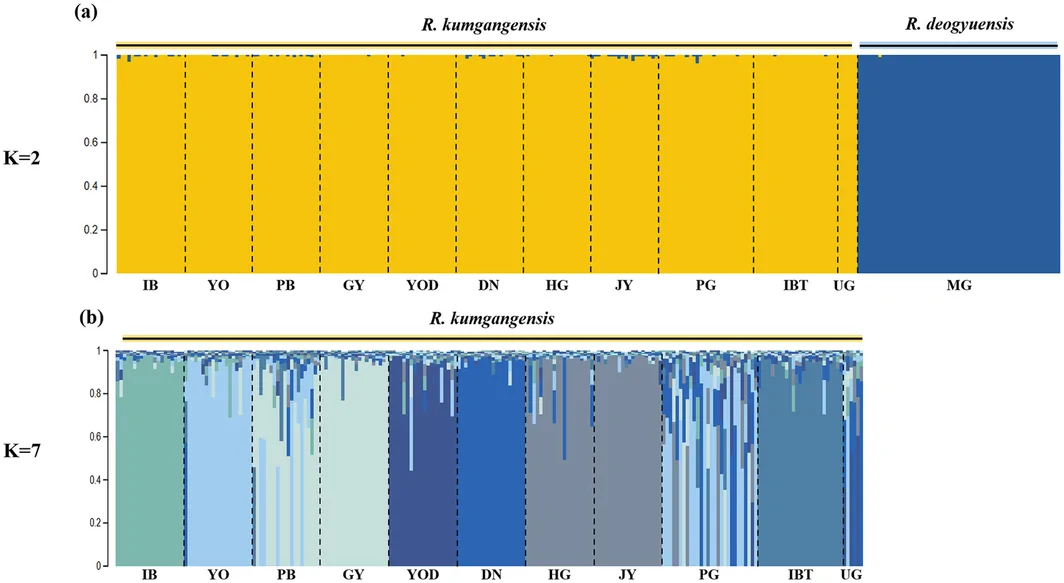
Population structure of *Rhynchocypris kumgangensis* and *R. deogyuensis* based on genotypes at eight microsatellite loci. Each individual is represented as a vertical bar partitioned into segments of different colors according to the proportion of the genome belonging to each of the identified clusters by STRUCTURE software. (a) Analyses of population structure for all 12 populations respond to *K* = 2. (b) Results of the only *R. kumgangensis* populations assignment test assuming *K* = 7.

**FIGURE 5:**
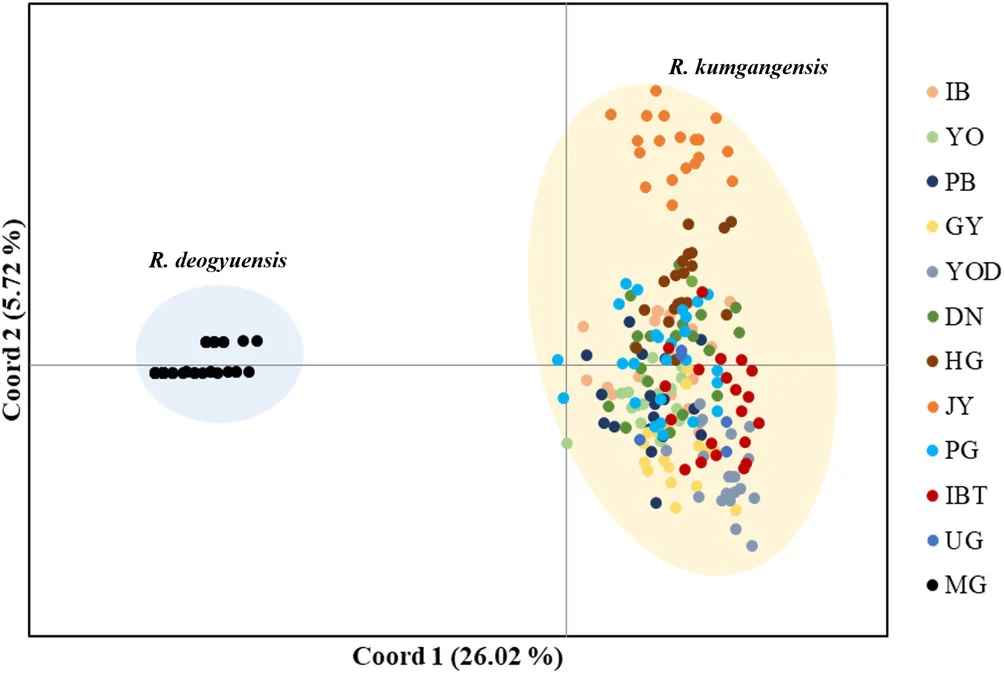
Results of principal coordinate analysis (PCoA) based on eight microsatellite genotypes for the 11 populations of *Rhynchocypris kumgangensis* and one population of *R. deogyuensis*.

STRUCTURE analyses based on microsatellites showed that 12 populations of *R. kumgangensis* and *R. deogyuensis* are most likely to form two genetically distinct clusters (*K* = 2), as revealed by a clear genetic clustering between *R. kumgangensis* and *R. deogyuensis* (Figure 4a). When only 11 populations of *R. kumgangensis* were analyzed for STRUCTURE, seven genetic clusters (*K* = 7) were identified (Figure 4b). The results of STRUCTURE HARVSTER analysis supporting the optimal *K* values for the full dataset, including both *R. kumgangensis* and *R. deogyuensis* are provided in Figure S1. Eleven populations of *R. kumgangensis* showed relatively distinct genetic structure for each local population, while a high level of admixture in the genetic structure can be observed for the PG population. These findings were consistent with the results of the highest genetic diversity for the PG population (Table 1). Similar to the results of STRUCTURE, the PCA of eight microsatellites also showed an apparent genetic divergence between *R. kumgangensis* and *R. deogyuensis* (Figure 5), suggesting that they are likely to be a genetically well‐separated distinct species. Individuals within the *R. deogyuensis* cluster showed slight dispersion along Coordinate 2, which explained 5.72% of the total variation. However, the level of this dispersion was much lower than within the *R. kumgangensis* cluster.

### 3.3 Phylogeographic Relationships and Divergence Time

**FIGURE 6:**
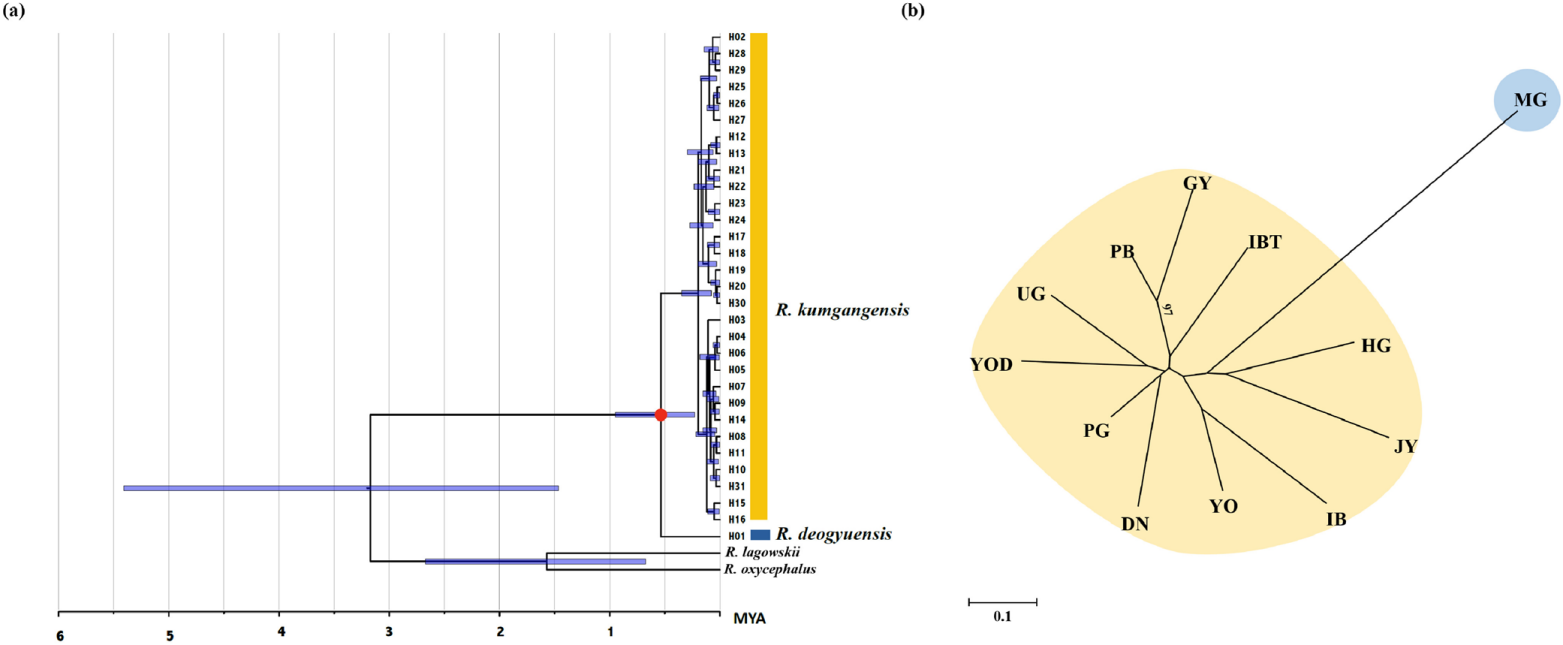
(a) Time‐calibrated phylogenetic tree showing divergence time estimates among haplotypes of *Rhynchocypris kumgangensis* and *R. deogyuensis* based on mitochondrial COI and CR sequences (1256 bp). Blue bars at nodes indicate 95% highest posterior density (HPD) intervals for divergence time estimates, and the red circle denotes the divergence between the two species. Time scale is shown in million years ago (MYA). (b) Phylogenetic relationships among 12 populations based on eight microsatellite loci for *R. kumgangensis* and *R. deogyuensis*.

The results of mtDNA phylogenetic analyses clearly distinguished the groups of *R. kumgangensis* and *R. deogyuensis* populations (Figure 6a). The populations of *R. kumgangensis* formed a monophyletic lineage, whereas the population of *R. deogyuensis* formed a well‐separated clade, supporting again that they are distinct species from a “phylogenetic” perspective. The results were further validated from microsatellite‐based neighbor‐joining trees (Figure 6b).

Divergence time estimates for *R. kumgangensis* and *R. deogyuensis* are shown in Figure 6a. Bayesian analyses suggest that the two species diverged during the middle Pleistocene, approximately 0.54 Mya [95% HPD (highest posterior density) = 0.23–0.95 Mya], corresponding to glacial–interglacial climatic fluctuations that may have influenced their evolutionary divergence.

### 3.4 Comparative Analysis of Mitogenomes

**FIGURE 7:**
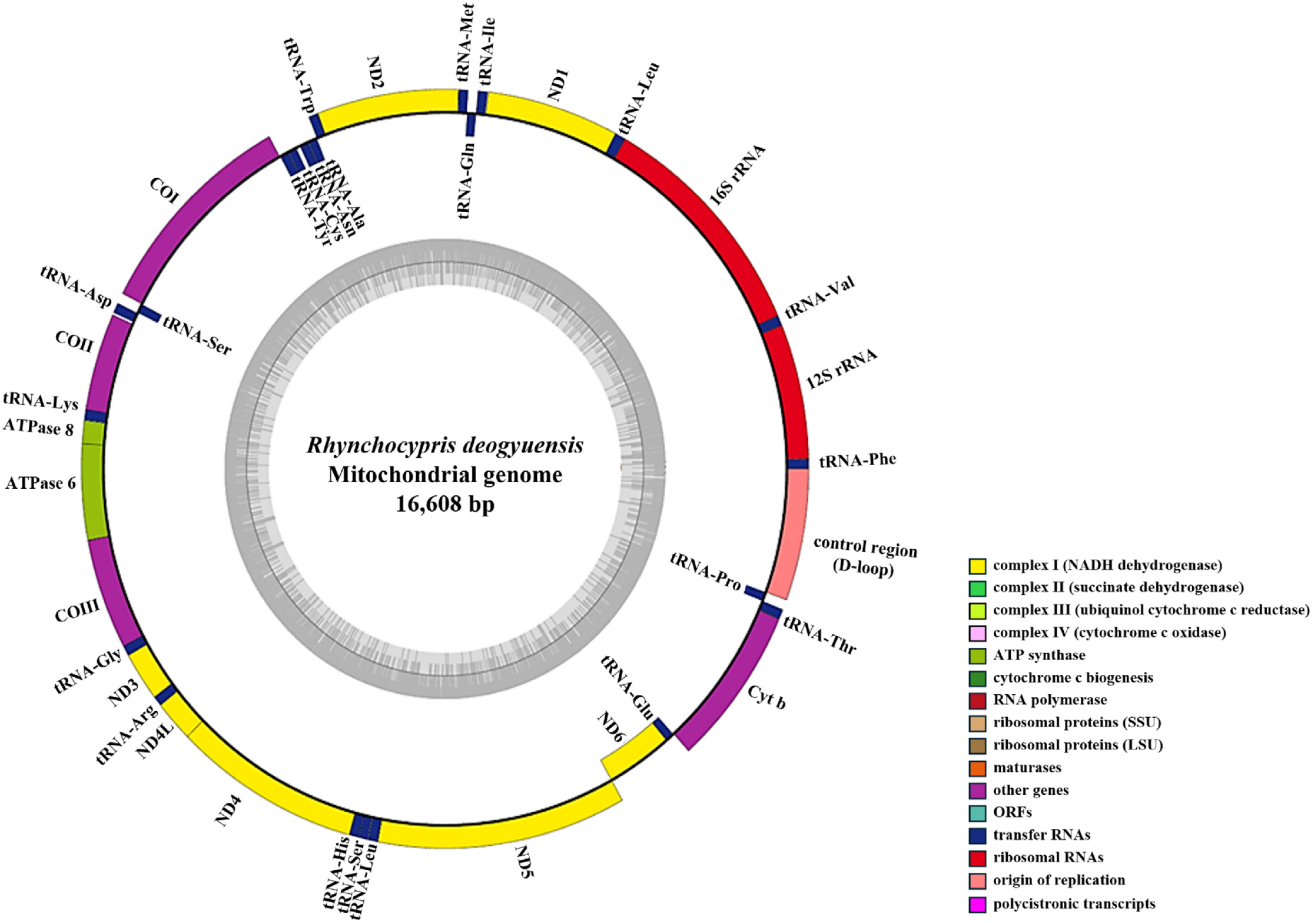
Map of the mitogenome of *Rhynchocypris deogyuensis*. Genes belonging to different functional groups are colored code. The dark‐gray inner circle represents GC content, and the light‐gray inner circle represents AT content.

The assembled mitogenome of *R. deogyuensis* showed a length of 16,608 bp and an overall GC content of 45.4%, and a total nucleotide composition of A—28.5%, C—27.5%, G—17.9% and T—26.1%. It consists of 13 protein coding genes (PCGs), 22 tRNA genes and 2 ribosomal RNA (rRNA) genes (Figure 7). By comparing the sequences between the mitochondrial genomes of *R. kumgangensis* (*N* = 2) and *R. deogyuensis* (*N* = 3), the number and order of genes were observed to be the same as the reference genomes, suggesting no structural variation such as insertions or rearrangements exists. When comparing the published available mitogenome database of *R. kumgangensis* with the analyzed mitogenomes of *R. deogyuensis*, an approximate difference of 2.9% was observed, suggesting that they showed the species‐level sequence divergence (Hebert et al. 2003). While a mean of 0.5% sequence divergence was found for two mitogenomes of *R. kumgangensis*, only an approximate 0.01% was observed for three genomes of *R. deogyuensis*, indicating an extremely low degree of mtDNA variation within *R. deogyuensis*.

### 3.5 Length–Weight Relationship and Condition Factor

**FIGURE 8:**
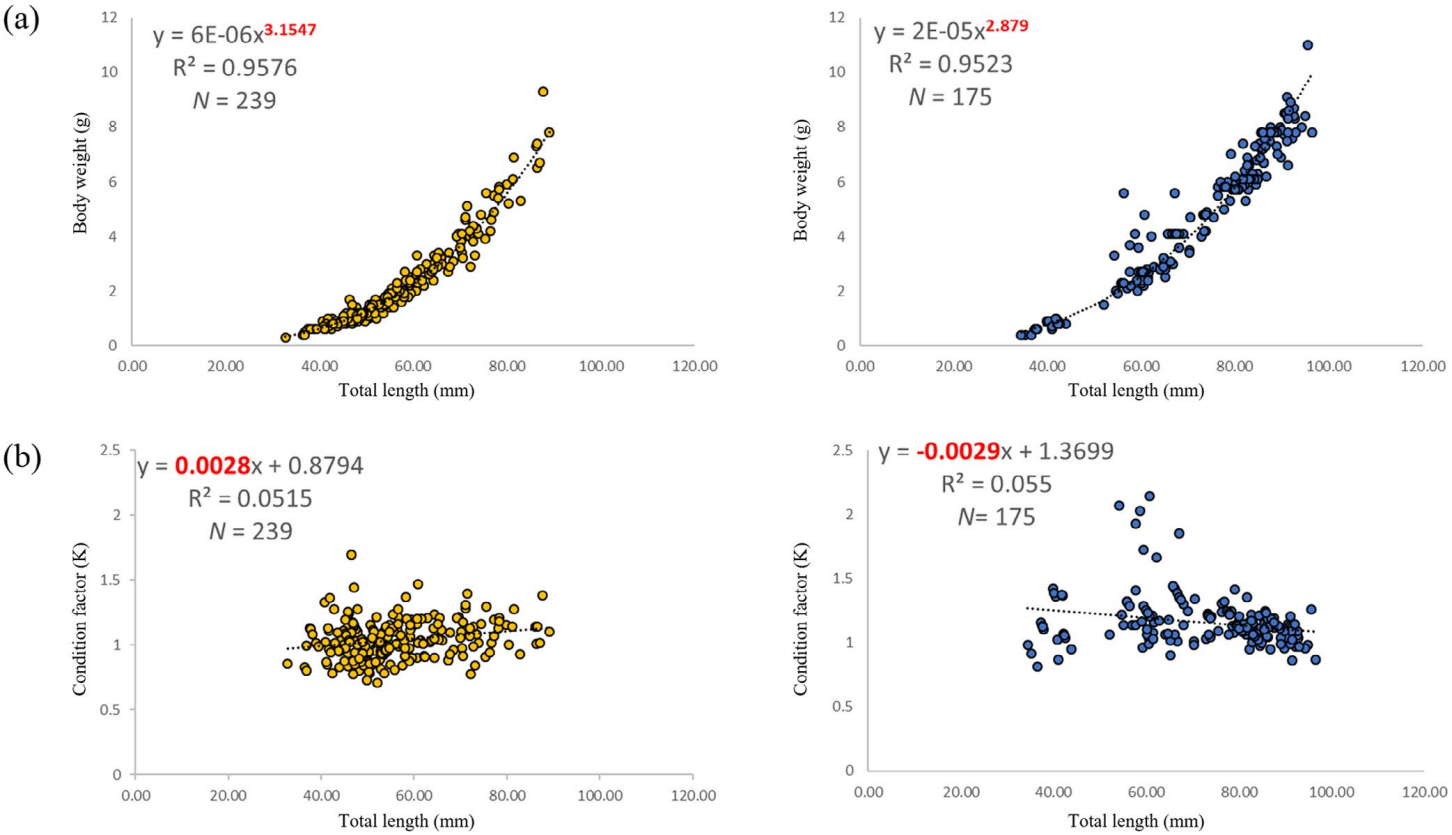
Length–weight relationship and condition factor of *Rhynchocypris kumgangensis* and *R. deogyuensis*. (a) Length–weight relationship of *R. kumgangensis* (left) and *R. deogyuensis* (right). (b) Condition factor of *R. kumgangensis* (left) and *R. deogyuensis* (right).

To determine the growth status and health conditions (e.g., fat content) of *R. kumgangensis* and *R. deogyuensis*, the length–weight relationship (LWR) and condition factor (*K*) were estimated for the populations collected before and after the spawning season from 2022 to 2023. In the LWR, when the parameter “*b*” is less than 3.0, that is, regression coefficients of LWR are < 3.0, the increase in weight is proportionally lower than that in length, indicating a relatively poor nutritional condition (Anderson and Gutreuter 1983). However, when “*b*” > 3.0, the growth pattern is positively allometric, meaning that weight increases at a faster rate than length, indicative of good body condition. *R. kumgangensis* showed *b* > 3.0 (3.155), whereas *R. deogyuensis* indicated *b* < 3.0 (2.879) (Figure 8a). With respect to the condition factor, the slope of *K* value was positive for *R. kumgangensis* (*K* = 0.0028), indicating that nutritional status increased with increasing body length. In contrast, the slope for *R. deogyuensis* was negative (*K* = –0.0029), showing a lower increase in body weight relative to growth, indicative of poor body condition (Figure 8b) (Jin et al. 2015).

### 3.6 Differences in Morphometric (Metric) Traits

The ratios of the measured morphometric traits of *R. kumgangensis* and *R. deogyuensis* to standard length (SL), expressed as percentages of SL (% SL), were calculated for head length (HL), body depth (BD), predorsal length (PDL), preanal length (PA), prepelvic length (PPL), and caudal peduncle depth (CPD) (Table 3). The ratio of eye diameter (ED) to head length (HL), expressed as a percentage of HL (% HL), was also calculated (Table 3). Among these seven traits, only four traits, including PA, PPL, CPD, and ED, differed significantly between *R. kumgangensis* and *R. deogyuensis* (*t*‐test, *p* < 0.05; Table 3). PA and PPL were significantly lower in *R. deogyuensis* [PA: 60.3–64.8 (62.4 ± 1.3) % SL; PPL: 40.2–47.7 (44.4 ± 1.5)] than in *R. kumgangensis* [PA: 60.0–67.7 (63.5 ± 1.6); PPL: 42.4–51.5 (45.9 ± 1.7)] (Table 3). In contrast, CPD was significantly higher in *R. deogyuensis* [10.2–11.9 (11.1 ± 0.4)] than in *R. kumgangensis* [8.5–11.4 (10.0 ± 0.6)] (*p* < 0.001). However, the ratio of ED to HL was significantly lower in *R. deogyuensis* [22.3–28.6 (26.2 ± 1.4)] than in *R. kumgangensis* [23.7–35.4 (30.4 ± 2.5)] (*p* < 0.001). The remaining traits, HL, BD, and PDL, did not differ significantly between the two species (*t*‐test, *p* > 0.05; Table 3).

**TABLE 3:** Comparisons of morphometric (metric) traits between *R. kumgangensis* and *R. deogyuensis*. Four of the seven traits (PA, PPL, CPD, and ED) were significantly different between *R. kumgangensis* and *R. deogyuensis* (*t*‐tests, *p* < 0.05).

Character	*Rhynchocypris kumgangensis*	*Rhynchocypris deogyuensis*	*p*‐values
No. of specimens	60	30	
Standard length (mm)	38.3–79.1	71.2–86.0	
In % of standard length			
Head length (HL)	18.1–25.5 (21.2 ± 1.6)	19.6–22.9 (21.2 ± 1.0)	0.809
Body depth (BD)	17.9–25.6 (21.3 ± 1.9)	20.1–23.8 (21.9 ± 1.0)	0.074
Predorsal length (PDL)	48.3–54.7 (51.7 ± 1.4)	48.1–55.5 (51.7 ± 1.4)	0.902
Preanal length (PA)	60.0–67.7 (63.5 ± 1.6)	60.3–64.8 (62.4 ± 1.3)	**0.002**
Prepelvic length (PPL)	42.4–51.5 (45.9 ± 1.7)	40.2–47.7 (44.4 ± 1.5)	**< 0.001**
Caudal peduncle depth (CPD)	8.5–11.4 (10.0 ± 0.6)	10.2–11.9 (11.1 ± 0.4)	**< 0.001**
In % of head length			
Eye diameter (ED)	23.7–35.4 (30.4 ± 2.5)	22.3–28.6 (26.2 ± 1.4)	**< 0.001**

*Note:* *p*‐values in bold indicate statistically significant differences (*p* < 0.05).

### 3.7 Geometric Morphometrics

The DFA analysis indicated significant morphological differences between *R. kumgangensis* and *R. deogyuensis*. Based on body shape, as measured by partial warps, DFA correctly classified individuals of *R. kumgangensis* and *R. deogyuensis*, respectively (*p* < 0.001 at 1000 permutations) (Figure 2b). In other words, shape assignments of *R. kumgangensis* and *R. deogyuensis* to their own morphological groups were 100% and 100%, respectively, using the discriminant function classification (Procrustes distance = 0.0218, *p* < 0.001). The CVA analysis of geometric shape data showed significant differences among the three populations (PG and PH of *R. kumgangensis*, and MG of *R. deogyuensis*) (Figure 2c). The relationships between the Procrustes distances, based on the superimpose shape data on size (standard length), were statistically significant (*p* < 0.001 at 1000 permutations) for the entire data set. Canonical variate 1 (CV1), which explained 71.03% of the total variance, clearly separated Cluster 1 (PH, PG) and Cluster 2 (MG) by a change in the position of the body shape related to body depth. In addition, CV2 accounted for 28.97% of the total variance observed, separating the PG and PH population. The MG population of *R. deogyuensis* was not separated in terms of the CV2.

## 4 Discussion

### 4.1 Population Genetic Structure Between *R. kumgangensis* and *R. deogyuensis*

To our best knowledge, this is the first study to document the degree of genetic diversity and geographic population structure of the Korean endemic cold‐water fishes *R. kumgangensis* and *R. deogyuensis* using multiple genetic markers, including mtDNA, microsatellites, and entire mitogenomes. The genetic analyses reveal that *R. deogyuensis* had substantially a lower level of genetic diversity than *R. kumgangensis*, as indicated by the presence of only a single matrilineal lineage (i.e., only one mtDNA haplotype detected; H01) and approximately 3.6 times as low in AR for *R. deogyuensis* (AR = 1.705) relative to *R. kumgangensis* (mean AR = 6.118) (Table 1). The findings are consistent with the previous results of twenty‐six haplotypes observed for *R. kumgangensis* populations from the Han River but only one haplotype for *R. deogyuensis* from the Geum River, based on mtDNA cytochrome *b* (cyt *b*; 1141 bp) sequences (Lee and Kim 2016; Lee and Sim 2017). The level of genetic diversity is known to be positively correlated with effective population size (*N*_e_) (Frankham 1996). Therefore, it would be conceivable that the *N*_e_ of the MG population of *R. deogyuensis*, characterized by markedly depleted genetic diversity, is considerably smaller than much more genetically diverse *R. kumgangensis* populations. The lower *N*_e_ estimate for *R. deogyuensis* (median = 30.1) compared to *R. kumgangensis* (*N*_e_ = 426.2 after excluding populations with *N*_e_ = ∞) seems to support this hypothesis. The LD‐based *N*_e_ estimates provide useful comparative insights into population size and genetic diversity levels, although future studies using genome‐wide markers or temporal sampling would help validate and refine these estimates. *Rhynchocypris kumgangensis* is predominantly distributed within the Han River basin located at the north‐eastern part of South Korea; however, *R. deogyuensis* only occurs at the Muju Gucheondong Valley from the Geum River basin in the Mount Deogyu National Park, which is located at the southwestern part of mainland. Given that both species are cold‐water fishes restricted to upstream habitats with temperatures below 20°C, the low genetic diversity observed in *R. deogyuensis* at its southernmost range boundary is likely attributable to its limited geographic distribution (Song 2000). The results of genetic diversity for *R. kumgangensis* revealed the lowest diversity in HG population, with only one haplotype detected. The field survey conducted in 2022 recorded only two *R. kumgangensis* individuals among 468 individuals representing seven species, accounting for 0.43% of the total number of individuals recorded, and supporting the notion of a low relative abundance in the HG region (Ministry of Environment [ME] 2022). Unexpectedly, despite its small population with limited distributional range, the UG population, which is the most distantly located, have a moderate level of microsatellite diversity with a high *N*_e_, perhaps resulting from a high degree of admixture or introgression between genetically divergent groups (Figure 4). It is worthy to trace the origin of the UG population using more samples as well as more markers, if necessary, given this population being introduced from elsewhere artificially.

Analyses of population structure and genetic differentiation, based on mtDNA and microsatellite data, reveal pronounced genetic divergence between *R. kumgangensis* and *R. deogyuensis* (Figure 4a), substantiating their status as distinct species. Genetic differentiation between the two species was consistently significant, with *F*_ST_ values ranging from 0.956 to 1.000 for mtDNA and from 0.536 to 0.711 for microsatellite markers. Comparable patterns of strong genetic differentiation have also been reported in another Korean endemic freshwater fish, the Korean dark chub (*Z. koreanus*). In that study, pairwise inter‐riverine *F*_ST_ values within *Z. koreanus* ranged from −0.01 to 0.96 for mtDNA and from 0.00 to 0.35 for microsatellites, whereas inter‐species *F*_ST_ values between *Z. koreanus* and its relative *Zacco temminckii* ranged from 0.66 to 0.85 for mtDNA and from 0.33 to 0.48 for microsatellites (Kim et al. 2026). These results indicate that the strong population structure observed in *Z. koreanus* is likely driven by geographic isolation among river basins.

The observed levels of genetic differentiation between *R. kumgangensis* and *R. deogyuensis* were higher than those reported for benthic and limnetic sticklebacks (*F*_ST_ = 0.209–0.336), which correspond to state 3, characterized by “strong but reversible reproductive isolation” (Hendry et al. 2009; Lee et al. 2025). Although some within‐species mtDNA *F*_ST_ values in *R. kumgangensis* were also high, reaching 0.923, microsatellite data showed a clearer distinction between intraspecific and interspecific genetic differentiation. Consequently, the two geographically isolated minnow groups (Kumgang and Deogyu fat minnows) may represent an advanced stage of divergence along the speciation continuum, potentially approaching state 4 of “complete and irreversible reproductive isolation” (Hendry et al. 2009). Future mating experiments will be useful for further evaluating the extent of reproductive isolation between *R. kumgangensis* and *R. deogyuensis*. The extremely high degrees of mitochondrial and nuclear divergence between the two species support the hypothesis that they are independent species (Lee and Sim 2017). When comparing the mitogenomes between the two species, *R. kumgangensis* exhibits a length of 16,604 bp, while *R. deogyuensis* shows a 4 bp longer mitogenome, resulting in an overall sequence divergence of approximately 2.9%. According to a previous work, in general, genetic divergence exceeding the threshold of 2%–3% has been suggested to be suitable for determining species boundaries in a majority of animals (Hebert et al. 2003; Kang et al. 2020; Jang et al. 2021; Kim et al. 2026). The whole mitogenome‐level genetic distance between *R. kumgangensis* and *R. deogyuensis* appears to be lower than or similar to that of a single mtDNA cyt *b* gene (2.9%–3.6%) examined in a previous study (Lee and Sim 2017). Nevertheless, when comparing the mitogenomes of three *R. deogyuensis* individuals, very low polymorphisms of 0.01% (SNP [Single Nucleotide Polymorphisms] = 3) were observed. These findings again underscore the low diversity within *R. deogyuensis* and emphasize the urgent need for conservation/restoration and management efforts for this particularly unique evolutionary legacy in Korea. Low genetic diversity may pose challenges for adapting to environmental changes, and species with higher genetic diversity are at a lower risk of extinction compared to those with reduced genetic variability (Markert et al. 2010; Martinez et al. 2018). Low genetic diversity may reduce the adaptive potential of *R. deogyuensis* under accelerating climate change. However, any intervention aimed at increasing genetic diversity should be considered cautiously after evaluating potential risks, including hybridization, loss of genetic identity, and loss of local adaptation.

### 4.2 Evolutionary Relationship Between *R. kumgangensis* and *R. deogyuensis*

The results of mtDNA and microsatellite‐based phylogenetic analyses showed that *R. kumgangensis* populations formed a single monophyletic clade, whereas *R. deogyuensis* represented a separate lineage. This pattern is consistent with previous findings based on mtDNA cyt *b* phylogeny (Lee and Kim 2016; Lee and Sim 2017). With increasing sample sizes and more molecular markers used in the current analyses, the results align with the previous findings, strongly suggesting that the eleven *R. kumgangensis* populations are composed of recently diversified a single clade, while *R. deogyuensis* population is comprised of a unique lineage (Figure 6).

The haplotype network analysis reveals a total of 31 haplotypes for *R. kumgangensis* and *R. deogyuensis*, with all 60 individuals in the *R. deogyuensis* population sharing a single haplotype (H01). Haplotype H01 exhibited a divergence of 22 mutations from the closest haplotype, H22 (PG; *R. kumgangensis*), indicating a distinct lineage in both phylogeny and network analysis. Interestingly and ironically, the matrilineal lineage of *R. deogyuensis* has evolved from the PG population of *R. kumgangensis*, representing its largest population in South Korea, although they are geographically quite distantly located. Within *R. kumgangensis*, a range of one to 10 mutations among the haplotypes is present, indicating its shallow evolutionary history, with H08 (13.3%) and H29 (19.6%) being likely the most ancestral matrilineal lineages for this species (Figure 3).

Our divergence time estimates suggest that *R. kumgangensis* and *R. deogyuensis* have diverged during the Pleistocene (0.23–0.95 Mya), consistent with divergence time estimates reported for other freshwater fishes endemic to the Korean Peninsula (Jeon et al. 2018, 2022). During this period, repeated glacial–interglacial cycles drove substantial fluctuations in sea level, which likely altered hydrological connectivity among river systems (Jeon et al. 2018; Shelley et al. 2020). This geological process may have led to the evolution of *R. deogyuensis* via allopatric speciation; however, this hypothesis requires further validation (Kim et al. 2026).

### 4.3 Morphological Differentiation Between *R. kumgangensis* and *R. deogyuensis*

Changes in body shape can rapidly integrate adaptive responses to diverse habitat environments, thereby reflecting ecological differentiation among populations (Burns et al. 2009; Fruciano et al. 2011; Stokes et al. 2025). Therefore, inter‐ and intra‐specific morphological divergence can elucidate adaptive potentials within and between species, which is particularly true for recently diverged populations or species (Stokes et al. 2025). According to previous research, there were no clear differences in morphological traits between *R. kumgangensis* and *R. deogyuensis*, with variations observed only in some metric traits, such as interorbital width, eye diameter (ED), caudal peduncle depth (CPD), number of scales above lateral line, and the presence of a black transverse bar on the first unbranched ray of the dorsal fin (Lee and Sim 2017). In this study, among the seven morphometric (metric) traits measured, four traits differed significantly between *R. kumgangensis* and *R. deogyuensis*. *Rhynchocypris kumgangensis* showed significantly higher PA, PPL, and ED, whereas *R. deogyuensis* showed significantly greater CPD (Table 3), indicating detectable morphological differentiation between the two species. These interspecific differences in morphological traits can be interpreted as evidence of morphological adaptation to microhabitat variation. For example, the relatively large eyes observed in *R. kumgangensis* may represent an adaptive trait for enhancing visual information detection in low light environments, likely reflecting an ecological response to limited light availability (Land and Nilsson 2012; Caves et al. 2017). Additionally, the increased prepelvic length may serve as a functional trait contributing to swimming stability or posture maintenance (Yamanoue et al. 2010; Ekstrom and Kajiura 2014). In contrast, the deeper caudal peduncle depth observed in *R. deogyuensis* may reflect an adaptation to fast flowing environments (Langerhans 2010). These four traits are considered sufficient to distinguish *R. kumgangensis* from *R. deogyuensis*. However, the comparison was based on only seven traits; additional morphological and meristic characters may be required.

Geometric morphometric analysis based on body shape reveals significant morphological differentiation between *R. kumgangensis* and *R. deogyuensis* (Figure 2). According to the results of DFA, *R. kumgangensis* and *R. deogyuensis* were morphologically distinguished with 100% accuracy, and there was no representation of an intermediate body shape between the two species, which strongly suggests that they evolve divergent morphology in response to their different environments. The differences in hydraulic environmental habitat conditions may account for body shape divergence between the species and among the populations that we observed (Drinan et al. 2012). Morphological traits related to body shape in freshwater fishes (e.g., body depth, caudal fin form) are found to be typically associated with flow velocity (Langerhans and Reznick 2010; Drinan et al. 2012) and therefore swimming capacity (Webb 1978; Gatz 1979). A more slender (fusiform) body (low RH) tends to confer higher fitness under higher velocity environments (Langerhans and Reznick 2010). A more streamlined body facilitates steady swimming in high‐flow environments by reducing the energetic cost of maintaining position in the water column, whereas a deeper body facilitates in low‐flow environments (Webb 1978). Additionally, differences were observed in eye diameter and snout length between *R. kumgangensis* and *R. deogyuensis*. These findings suggest ongoing morphological differentiation between the two species.

### 4.4 Differences in Ecological Conditions Between *R. kumgangensis* and *R. deogyuensis*

Fish's fat content and condition factor can serve as crucial indicators for evaluating their health condition and reproductive capacity in a given environment, offering valuable insights into habitat quality, feeding efficiency, and other pertinent ecological factors (Anderson and Gutreuter 1983). Regarding the LWR, the estimated parameter *b*‐value for *R. deogyuensis* was 2.879, which is lower than 3.0, suggesting that the growth rates, nutritional status, and body condition are generally poor (Jin et al. 2015). The observed unhealthy body condition for *R. deogyuensis* is perhaps associated with the very low genetic diversity at least in part resulting from high levels of inbreeding (*F*_IS_ = 0.248). In contrast, the parameter *b*‐values for two *R. kumgangensis* populations were found to be 3.155. Typically, when the parameter *b* is greater than 3.0, it indicates that individuals are disproportionately large relative to their length (Seo 2005), suggesting that the health condition and reproductive capacity are generally in good condition.

The condition factor slope (*K*) signifies the rate of weight gain relative to length, where a positive value denotes a rapid weight increase and a negative value indicates a slower increase (Anderson and Neumann 1996). *Rhynchocypris kumgangensis* populations demonstrated a positive value of 0.0028, whereas the *R. deogyuensis* population exhibited a negative value of −0.0029. These findings indicate that *R. kumgangensis* populations are well‐adapted to the environment, with a relatively favorable nutritional status, while conversely, the *R. deogyuensis* population is determined to have a poor nutritional status, again probably due to the combined effects of inbreeding and depleted genetic diversity. The low nutritional status for *R. deogyuensis* could also be a result of strong predation by introduced species such as *Oncorhynchus mykiss* , which does not originally inhabit there (Lee 2020; Hwang et al. 2026).

In conclusion, several lines of multifaceted evidence—substantial population structure and genetic differentiation, the inter‐species level divergence in the mitochondrial genomes, distinct phylogenetic clusters, and significant morphological divergence between *R. kumgangensis* and *R. deogyuensis*—all strongly suggest that they are likely to be separate species. However, determining the “stage” of speciation processes should be studied further. For example, it is suggested that historical demography analysis is necessary to track events that occurred in populations of both species at specific times in the past and to understand the migration history of the two species (Jeon et al. 2018). From a conservation biology perspective, the extremely low genetic diversity, elevated inbreeding, and poor health and ecological conditions of *R. deogyuensis* highlight an urgent need for conservation/restoration and management efforts with a high priority.

## Author Contributions

**Soon Young Hwang:** data curation (lead), formal analysis (lead), investigation (lead), visualization (lead), writing – original draft (lead), writing – review and editing (equal). **Ji Eun Jang:** data curation (supporting), funding acquisition (lead), writing – review and editing (equal). **Ji Hyoun Kang:** formal analysis (lead), funding acquisition (lead), writing – original draft (lead). **Hyuk Je Lee:** conceptualization (lead), funding acquisition (lead), supervision (lead), writing – original draft (lead), writing – review and editing (lead).

## Funding

This study was supported by a grant from the Korea National Park Research Institute (Project Number: NPRI2022‐05), Korea National Park Service, by the National Research Foundation (NRF) grant funded by the Korea government (MSIT) (RS‐2024‐00350469), Korean Institute of Marine Science and Technology Promotion (KIMST) funded to the Ministry of Oceans and Fisheries, Korea (RS‐2025‐02304428), and also partly by Graduate School of the Sangji University.

## Conflicts of Interest

The authors declare no conflicts of interest.

## Supporting information


**Figure S1:** Results of STRUCTURE HARVESTER analysis base on the Evanno Δ*K* method for the full dataset including *R. kumgangensis* and *R. deogyuensis*. The highest Δ*K* value was observed at *K* = 2, supporting two major genetic clusters corresponding to *R. kumgangensis* and *R. deogyuensis*. The red vertical line indicates the optimal *K* value.

**Table S1:** Information of sampling localities, river basins, location codes and latitude/longitude in this study.

**Data S1:** ece374253‐sup‐0003‐Supinfo.xlsx.

## Data Availability

Mitochondrial haplotype sequences and three mitogenome sequences obtained for this study have been deposited in GenBank under the accession numbers PZ298828−PZ298858, PZ304178−PZ304208, and PZ267738–PZ267740. All the required data are uploaded as Supporting Information.

## References

[ece374253-bib-0001] Abràmoff, M. D. , P. J. Magalhães , and S. J. Ram . 2004. “Image Processing With ImageJ.” Biophotonics International 11: 36–42. http://hdl.handle.net/1874/204900.

[ece374253-bib-0002] Adeoba, M. I. , R. Kabongo , H. Van der Bank , and K. Yessoufou . 2018. “Re‐Evaluation of the Discriminatory Power of DNA Barcoding on Some Specimens of African *Cyprinidae* (Subfamilies *Cyprininae* and *Danioninae*).” ZooKeys 746: 105–121. 10.3897/zookeys.746.13502. PMC5906743 29674898

[ece374253-bib-0003] Alekseyev, S. S. , N. V. Gordeeva , V. P. Samusenok , et al. 2023. “Profound and Rapid Allopatric Differentiation of Arctic Charr *Salvelinus alpinus* on a Microgeographic Scale.” Hydrobiologia 850: 1021–1043. 10.1007/s10750-022-05064-8.

[ece374253-bib-0004] Anderson, R. O. , and S. J. Gutreuter . 1983. “Length, Weight, and Associated Structural Indices.” In Fisheries Techniques, edited by L. A. Nielsen and D. L. Johnson , 283–300. American Fisheries Society.

[ece374253-bib-0091] Anderson, R. O. , and R. M. Neumann . 1996. “Length, Weight, and Associated Structural Indices.” In Fisheries Techniques, edited by B. R. Murphy and D. W. Willis , 2nd ed., 447–482. American Fisheries Society.

[ece374253-bib-0005] April, J. , R. H. Hanner , A. M. Dion‐Côté , and L. Bernatchez . 2013. “Glacial Cycles as an Allopatric Speciation Pump in North‐Eastern A Merican Freshwater Fishes.” Molecular Ecology 22: 409–422. 10.1111/mec.12116. 23206322

[ece374253-bib-0006] Arai, R. , S. R. Jeon , and T. Ueda . 2001. “ *Rhodeus pseudosericeus* sp. nov., a New Bitterling From South Korea (Cyprinidae, Acheilognathinae).” Ichthyological Research 48: 275–282. 10.1007/s10228-001-8146-1.

[ece374253-bib-0007] Arthington, A. H. , N. K. Dulvy , W. Gladstone , and I. J. Winfield . 2016. “Fish Conservation in Freshwater and Marine Realms: Status, Threats and Management.” Aquatic Conservation: Marine and Freshwater Ecosystems 26: 838–857. 10.1002/aqc.2712.

[ece374253-bib-0008] Baek, H. M. , H. B. Song , H. S. Sim , Y. G. Kim , and O. K. Kwon . 2002. “Habitat Segregation and Prey Selectivity on Cohabitation Fishes, *Phoxinus phoxinus* and *Rhynchocypris kumgangensis* .” Korean Journal of Ichthyology 19: 59–69.

[ece374253-bib-0009] Burns, J. G. , N. P. Di , and F. H. Rodd . 2009. “The Role of Predation in Variation in Body Shape in Guppies *Poecilia reticulata* : A Comparison of Field and Common Garden Phenotypes.” Journal of Fish Biology 75: 1144–1157. 10.1111/j.1095-8649.2009.02314.x. 20738605

[ece374253-bib-0010] Byeon, H. K. 2016. “The First Record on the *Rhynchocypris kumgangensis* From Taehwa River, Korea.” Korean Journal of Environment and Ecology 30: 745–750. 10.13047/KJEE.2016.30.4.745.

[ece374253-bib-0011] Caves, E. M. , T. T. Sutton , and S. Johnsen . 2017. “Visual Acuity in Ray‐Finned Fishes Correlates With Eye Size and Habitat.” Journal of Experimental Biology 220: 1586–1596. 10.1242/jeb.151183. 28183870

[ece374253-bib-0089] Choi, H. K. , and H. J. Lee . 2025. “Genetic Diversity and Phylogeographic Structure of the Endangered Korean Bitterling (*Tanakia Signifer*) and Its Symbiotic Mussel (*Nodularia Breviconcha*), Based on Mitochondrial DNA Variation: Implications for Conservation.” Conservation Genetics 26: 513–528. 10.1007/s10592-025-01685-3.

[ece374253-bib-0012] Choi, K. C. 1973. “On the Geographical Distribution of Freshwater Fishes South of DMZ in Korea.” Korean Journal of Limnology 6: 29–36.

[ece374253-bib-0013] Coyne, J. A. 1992. “Genetics and Speciation.” Nature 355: 511–515. 10.1038/355511a0. 1741030

[ece374253-bib-0014] Do, C. , R. S. Waples , D. Peel , G. M. Macbeth , B. J. Tillett , and J. R. Ovenden . 2014. “NeEstimator v2: Re‐Implementation of Software for the Estimation of Contemporary Effective Population Size (*N* _e_) From Genetic Data.” Molecular Ecology Resources 14: 209–214. 10.1111/1755-0998.12157. 23992227

[ece374253-bib-0015] Drinan, T. J. , P. McGinnity , J. P. Coughlan , T. F. Cross , and S. S. Harrison . 2012. “Morphological Variability of Atlantic Salmon Salmo Salar and Brown Trout *Salmo trutta* in Different River Environments.” Ecology of Freshwater Fish 21: 420–432. 10.1111/j.1600-0633.2012.00561.x.

[ece374253-bib-0016] Drummond, A. J. , M. A. Suchard , D. Xie , and A. Rambaut . 2012. “Bayesian Phylogenetics With BEAUti and the BEAST 1.7.” Molecular Biology and Evolution 298: 1969–1973. 10.1093/molbev/mss075. PMC3408070 22367748

[ece374253-bib-0017] Earl, D. A. , and B. M. VonHoldt . 2012. “STRUCTURE HARVESTER: A Website and Program for Visualizing STRUCTURE Output and Implementing the Evanno Method.” Conservation Genetics Resources 4: 359–361. 10.1007/s12686-011-9548-7.

[ece374253-bib-0018] Ekstrom, L. J. , and S. M. Kajiura . 2014. “Pelvic Girdle Shape Predicts Locomotion and Phylogeny in Batoids.” Journal of Morphology 275: 100–110. 10.1002/jmor.20201. 24142882

[ece374253-bib-0019] Excoffier, L. , and H. E. Lischer . 2010. “Arlequin Suite Ver 3.5: A New Series of Programs to Perform Population Genetics Analyses Under Linux and Windows.” Molecular Ecology Resources 10: 564–567. 10.1111/j.1755-0998.2010.02847.x. 21565059

[ece374253-bib-0020] Ficke, A. D. , C. A. Myrick , and L. J. Hansen . 2007. “Potential Impacts of Global Climate Change on Freshwater Fisheries.” Reviews in Fish Biology and Fisheries 17: 581–613. 10.1007/s11160-007-9059-5.

[ece374253-bib-0021] Frankham, R. 1996. “Relationship of Genetic Variation to Population Size in Wildlife.” Conservation Biology 10: 1500–1508. 10.1046/j.1523-1739.1996.10061500.x.

[ece374253-bib-0022] Fruciano, C. , C. Tigano , and V. Ferrito . 2011. “Geographical and Morphological Variation Within and Between Colour Phases in *Coris julis* (L. 1758), a Protogynous Marine Fish.” Biological Journal of the Linnean Society 104: 148–162. 10.1111/j.1095-8312.2011.01700.x.

[ece374253-bib-0023] Fulton, T. W. 1904. “The Rate of Growth of Fishes.” Twenty‐Second Annual Report 3: 141–241.

[ece374253-bib-0024] Gaston, K. J. , T. M. Blackburn , and J. H. Lawton . 1997. “Interspecific Abundance‐Range Size Relationships: An Appraisal of Mechanisms.” Journal of Animal Ecology 66: 579–601. 10.2307/5951.

[ece374253-bib-0025] Gatz, J. A. 1979. “Community Organization in Fishes as Indicated by Morphological Features.” Ecology 60: 711–718. 10.2307/1936608.

[ece374253-bib-0026] Goudet, J. 2001. “FSTAT, a Program to Estimate and Test Gene Diversities and Fixation Indices (Version 2.9. 3.2).” http://www2.unil.ch/popgen/softwares/fstat.htm.

[ece374253-bib-0027] Greiner, S. , P. Lehwark , and R. Bock . 2019. “OrganellarGenomeDRAW (OGDRAW) Version 1.3.1: Expanded Toolkit for the Graphical Visualization of Organellar Genomes.” Nucleic Acids Research 47: W59–W64. 10.1093/nar/gkz238. 30949694 PMC6602502

[ece374253-bib-0028] Harrison, I. , R. Abell , W. Darwall , M. L. Thieme , D. Tickner , and I. Timboe . 2018. “The Freshwater Biodiversity Crisis.” Science 362: 1369. 10.1126/science.aav9242. 30573621

[ece374253-bib-0029] Hebert, P. D. , A. Cywinska , S. L. Ball , and J. R. DeWaard . 2003. “Biological Identifications Through DNA Barcodes.” Proceedings of the Royal Society of London. Series B: Biological Sciences 270: 313. 10.1098/rspb.2002.2218. PMC1691236 12614582

[ece374253-bib-0030] Hendry, A. P. , D. I. Bolnick , D. Berner , and C. L. Peichel . 2009. “Along the Speciation Continuum in Sticklebacks.” Journal of Fish Biology 75: 2000–2036. 10.1111/j.1095-8649.2009.02419.x. 20738669

[ece374253-bib-0031] Hwang, S. Y. , H. K. Choi , Y. R. Kim , et al. 2026. “Ecological Characteristics of *Rhynchocypris deogyuensis* Inhabiting the Gucheondong Valley in the Deogyusan National Park: Its Population Size, Health Condition, and Predation Pressure.” Journal of National Park Research 17: 13–25. 10.54406/jnpr.2026.17.1.13.

[ece374253-bib-0032] Jalili, P. , S. Eagderi , and Y. Keivany . 2015. “Body Shape Comparison of Kura Bleak ( *Alburnus filippii* ) in Aras and Ahar‐Chai Rivers Using Geometric Morphometric Approach.” Research in Zoology 5: 20–24. 10.5923/j.zoology.20150501.03.

[ece374253-bib-0033] Jang, J. E. , S. Y. Byeon , H. R. Kim , J. Y. Kim , H. H. Myeong , and H. J. Lee . 2021. “Genetic Evidence for Sex‐Biased Dispersal and Cryptic Diversity in the Greater Horseshoe Bat, *Rhinolophus ferrumequinum* .” Biodiversity and Conservation 30: 847–864. 10.1007/s10531-021-02120-y.

[ece374253-bib-0034] Jeon, H. B. , D. Y. Kim , Y. J. Lee , H. G. Bae , and H. Y. Suk . 2018. “The Genetic Structure of *Squalidus multimaculatus* Revealing the Historical Pattern of Serial Colonization on the Tip of East Asian Continent.” Scientific Reports 8: 10629. 10.1038/s41598-018-28340-x. 30006507 PMC6045656

[ece374253-bib-0035] Jeon, H. B. , H. Y. Song , H. Y. Suk , and I. C. Bang . 2022. “Phylogeography of the Korean Endemic *Coreoleuciscus* (Cypriniformes: Gobionidae): The Genetic Evidence of Colonization Through Eurasian Continent to the Korean Peninsula During Late Plio‐Pleistocene.” Genes & Genomics 44: 709–719. 10.1007/s13258-022-01243-y. 35438462 PMC9120112

[ece374253-bib-0036] Jeon, H. J. , J. I. Baek , K. H. Kim , et al. 2019. “Community Structure of Fish and Distribution Characteristics of *Phoxinus phoxinus* and *Rhynchocypris kumgangensis* in the Gihwacheon Stream of Namhangang River, Korea.” Korean Journal of Ichthyology 31: 90–100. https://kiss.kstudy.com/Detail/Ar?key=3690391.

[ece374253-bib-0037] Jeon, Y. S. , M. H. Ko , E. D. Vasil'eva , R. Y. Myung , and Y. J. Won . 2020. “Genetic Patterns Reveal Northward Range Expansion and Cryptic Diversity in Nalbant's Spined Loach, *Cobitis nalbanti* Sensu *lato* (Teleostei: Cypriniformes: Cobitidae).” Systematics and Biodiversity 18: 1–11. 10.1080/14772000.2020.1737842.

[ece374253-bib-0038] Jin, S. , X. Yan , H. Zhang , and W. Fan . 2015. “Weight–Length Relationships and Fulton's Condition Factors of Skipjack Tuna ( *Katsuwonus pelamis* ) in the Western and Central Pacific Ocean.” PeerJ 3: e758. 10.7717/peerj.758. 25699207 PMC4330904

[ece374253-bib-0039] Kang, J. H. , J. E. Jang , J. H. Kim , et al. 2020. “The Origin of the Subtropical Coral *Alveopora japonica* (Scleractinia: Acroporidae) in High‐Latitude Environments.” Frontiers in Ecology and Evolution 8: 12. 10.3389/fevo.2020.00012.

[ece374253-bib-0040] Kim, D. M. , H. B. Jeon , and H. Y. Suk . 2014. “ *Tanakia latimarginata*, a New Species of Bitterling From the Nakdong River, South Korea (Teleostei: Cyprinidae).” Ichthyological Exploration of Freshwaters 25: 59–68.

[ece374253-bib-0041] Kim, I. S. , M. K. Oh , and K. Hosoya . 2005. “A New Species of Cyprinid Fish, *Zacco koreanus* With Redescription of *Z. temminckii* (Cyprinidae) From Korea.” Korean Journal of Ichthyology 17: 1–7.

[ece374253-bib-0042] Kim, I. S. , and Y. M. Son . 1984. “ *Cobitis choii* , A New Cobitid Fish From Korea.” Korean Journal of Zoology 27: 49–55.

[ece374253-bib-0043] Kim, S. , H. S. Noh , S. J. Hong , K. J. Kawak , and H. S. Kim . 2013. “Impact of Climate Change on Habitat of the *Rhynchocypris kumgangensis* in Pyungchang River.” Journal of Wetlands Research 15: 271–820. 10.17663/JWR.2013.15.2.271.

[ece374253-bib-0044] Kim, Y. R. , J. E. Jang , H. Choi , et al. 2026. “Cryptic Diversity and Genetic Variation in a Korean Endemic Freshwater Fish, the Korean Dark Chub (*Zacco koreanus*) Compared With Its Relative *Zacco temminckii* .” Ecology and Evolution 16: e73451. 10.1002/ece3.73451. 41958735 PMC13058722

[ece374253-bib-0045] Kim, Y. R. , J. E. Jang , H. K. Choi , and H. J. Lee . 2020. “Phylogeographic and Population Genetic Study of a Korean Endemic Freshwater Fish Species, *Zacco koreanus* .” Korean Journal of Environmental Biology 38: 650–657. 10.11626/KJEB.2020.38.4.650.

[ece374253-bib-0046] Klingenberg, C. P. 2011. “MorphoJ: An Integrated Software Package for Geometric Morphometrics.” Molecular Ecology Resources 11: 353–357. 10.1111/j.1755-0998.2010.02924.x. 21429143

[ece374253-bib-0047] Kumar, S. , G. Stecher , M. Li , C. Knyaz , and K. Tamura . 2018. “MEGA X: Molecular Evolutionary Genetics Analysis Across Computing Platforms.” Molecular Biology and Evolution 35: 1547–1549. 10.1093/molbev/msy096. 29722887 PMC5967553

[ece374253-bib-0048] Land, M. F. , and D. E. Nilsson . 2012. Animal Eyes. Oxford University Press.

[ece374253-bib-0049] Langerhans, R. B. 2010. “Predicting Evolution With Generalized Models of Divergent Selection: A Case Study With Poeciliid Fish.” Integrative and Comparative Biology 50: 1167–1184. 10.1093/icb/icq117. 21558265

[ece374253-bib-0050] Langerhans, R. B. , and D. N. Reznick . 2010. “Ecology and Evolution of Swimming Performance in Fishes: Predicting Evolution With Biomechanics.” In Fish Locomotion: An Eco‐Ethological Perspective, vol. 200, 200–248. CRC Press.

[ece374253-bib-0051] Lee, J. Y. , J. Choi , J. K. Kim , Y. S. Jang , K. Y. Lee , and B. Kim . 2008. “Ecological Effects of Kumgang Fat Minnow (*Rhynchocypris kumgangensis*) on Turbid Water.” Korean Journal of Environment and Ecology 22: 184–191.

[ece374253-bib-0052] Lee, J. Y. , K. H. Kim , H. J. Lee , S. G. Hwang , and T. S. Kang . 2025. “Genetic Variations Suggests That *Takifugu rubripes* , *T. chinensis*, and *T. pseudommus* Are the Same Species With a Shared Gene Pool.” Frontiers in Marine Science 11: 1506390. 10.3389/fmars.2024.1506390.

[ece374253-bib-0053] Lee, S. R. , and J. J. Kim . 2016. “Molecular Phylogeny Study of *Rhynchocypris kumkangensis* in Korea Based on the Mitochondrial DNA Cytochrom *b* Gene.” Journal of National Research 7: 147–153.

[ece374253-bib-0054] Lee, S. R. , and J. H. Sim . 2017. “A New Species of Deogyu Fat‐Minnow, *Rhynchocypris deogyuensis* (Cypriniformes: Cyprinidae) From Korea.” Journal of National Park Research 8: 1–7.

[ece374253-bib-0055] Lee, Y. G. 2020. “The Ecological Study of the Endemic Korean Fat Minnow, *Rhynchocypris kumgangensis* (Cyprinidae) in Deogyusan National Park.” Journal of National Park Research 11: 1–20.

[ece374253-bib-0056] Lee, Y. J. , H. G. Bae , H. B. Jeon , D. Y. Kim , and H. Y. Suk . 2017. “Human‐Mediated Processes Affecting Distribution and Genetic Structure of *Squalidus multimaculatus* , a Freshwater Cyprinid With Small Spatial Range.” Animal Cells and Systems 21: 349–357. 10.1080/19768354.2017.1371074.

[ece374253-bib-0057] Maderbacher, M. , C. Bauer , J. Herler , L. Postl , L. Makasa , and C. Sturmbauer . 2008. “Assessment of Traditional Versus Geometric Morphometrics for Discriminating Populations of the *Tropheus moorii* Species Complex (Teleostei: Cichlidae), a Lake Tanganyika Model for Allopatric Speciation.” Journal of Zoological Systematics and Evolutionary Research 46: 153–161. 10.1111/j.1439-0469.2007.00447.x.

[ece374253-bib-0058] Markert, J. A. , D. M. Champlin , R. Gutjahr‐Gobell , et al. 2010. “Population Genetic Diversity and Fitness in Multiple Environments.” BMC Evolutionary Biology 10: 1–13. 10.1186/1471-2148-10-205. 20609254 PMC2927917

[ece374253-bib-0059] Martinez, A. S. , J. R. Willoughby , and M. R. Christie . 2018. “Genetic Diversity in Fishes Is Influenced by Habitat Type and Life‐History Variation.” Ecology and Evolution 8: 12022–12031. 10.1002/ece3.4661. 30598796 PMC6303716

[ece374253-bib-0060] Ministry of Environment (ME) . 2022. “Water Environment Information System (WEIS).” http://water.nier.go.kr.

[ece374253-bib-0061] NIBR . 2010. National Climate Change Indicator Species of Korea [In Korea]. National Institute of Biological Resource.

[ece374253-bib-0062] Peakall, R. O. D. , and P. E. Smouse . 2006. “GENALEX 6: Genetic Analysis in Excel. Population Genetic Software for Teaching and Research.” Molecular Ecology Notes 6: 288–295. 10.1111/j.1471-8286.2005.01155.x. PMC3463245 22820204

[ece374253-bib-0063] Petit, R. J. , M. A. El , and O. Pons . 1998. “Identifying Populations for Conservation on the Basis of Genetic Markers.” Conservation Biology 12: 844–855. 10.1111/j.1523-1739.1998.96489.x.

[ece374253-bib-0064] Rahel, F. J. , C. J. Keleher , and J. L. Anderson . 1996. “Potential Habitat Loss and Population Fragmentation for Cold Water Fish in the North Platte River Drainage of the Rocky Mountains: Response to Climate Warming.” Limnology and Oceanography 41: 1116–1123. 10.4319/lo.1996.41.5.1116.

[ece374253-bib-0065] Reid, A. J. , A. K. Carlson , I. F.Creed , et al. 2019. “Emerging Threats and Persistent Conservation Challenges for Freshwater Biodiversity.” Biological Reviews 94: 849–873. 10.1111/brv.12480. 30467930

[ece374253-bib-0066] Rohlf, F. J. 1999. “Shape Statistics: Procrustes Superimpositions and Tangent Spaces.” Journal of Classification 16: 197–223. 10.1007/s003579900054.

[ece374253-bib-0067] Rohlf, F. J. 2015. “The Tps Series of Software.” Hystrix 26: 9–12. 10.4404/hystrix-26.1-11264.

[ece374253-bib-0068] Rohlf, F. J. , and L. F. Marcus . 1993. “A Revolution Morphometrics.” Trends in Ecology & Evolution 8: 129–132. 10.1016/0169-5347(93)90024-J. 21236128

[ece374253-bib-0069] Rousset, F. 2008. “genepop'007: A Complete Re‐Implementation of the Genepop Software for Windows and Linux.” Molecular Ecology Resources 8: 103–106. 10.1111/j.1471-8286.2007.01931.x. 21585727

[ece374253-bib-0070] Schindler, D. W. , K. G. Beaty , E. J. Fee , et al. 1990. “Effects of Climatic Warming on Lakes of the Central Boreal Forest.” Science 250: 967–970. 10.1126/science.250.4983.967. 17746921

[ece374253-bib-0071] Seehausen, O. , and C. E. Wagner . 2014. “Speciation in Freshwater Fishes.” Annual Review of Ecology, Evolution, and Systematics 45: 621–651. 10.1146/annurev-ecolsys-120213-091818.

[ece374253-bib-0072] Seo, J. W. 2005. “Fish Fauna and Ecological Characteristics of Dark Chub ( *Zacco temminckii* ) Population in the Mid‐Upper Region of Gam Stream.” Korean Journal of Ecology and Environment 38: 196–206.

[ece374253-bib-0073] Shelley, J. J. , S. E. Swearer , T. Dempster , et al. 2020. “Plio‐Pleistocene Sea‐Level Changes Drive Speciation of Freshwater Fishes in North‐Western Australia.” Journal of Biogeography 47: 1727–1738. 10.1111/jbi.13856.

[ece374253-bib-0074] Song, H. B. 2000. “Fat Minnow, Population Ecology of Fat Minnow, *Rhynchocypris kumgangensis* (Cyprinidae) in Korea.” Korean Journal of Ichthyology 12: 101–110.

[ece374253-bib-0075] Song, H. B. , and S. S. Choi . 1997. “Development of Eggs and Larvae of Korean Fatminnow, *Moroco kumgangensis* (Cyprinidae).” Korean Journal of Limnology 30: 67–74.

[ece374253-bib-0076] Stefan, H. G. , X. Fang , and J. G. Eaton . 2001. “Simulated Fish Habitat Changes in North American Lakes in Response to Projected Climate Warming.” Transactions of the American Fisheries Society 130: 459–477. 10.1577/1548-8659(2001)130<0459:SFHCIN>2.0.CO;2.

[ece374253-bib-0077] Stokes, M. F. , R. C. Harrington , D. Kim , et al. 2025. “The Role of Ecology in Allopatric Speciation of Darters in the Central Highlands, USA.” Evolution 80: 472–486. 10.1093/evolut/qpaf249. 41370042

[ece374253-bib-0078] Takezaki, N. , M. Nei , and K. Tamura . 2010. “POPTREE2: Software for Constructing Population Trees From Allele Frequency Data and Computing Other Population Statistics With Windows Interface.” Molecular Biology and Evolution 27: 747–752. 10.1093/molbev/msp312. 20022889 PMC2877541

[ece374253-bib-0079] Teacher, A. G. F. , and D. J. Griffiths . 2011. “HapStar: Automated Haplotype Network Layout and Visualization.” Molecular Ecology Resources 11: 151–153. 10.1111/j.1755-0998.2010.02890.x. 21429113

[ece374253-bib-0080] Van Oosterhout, C. , W. F. Hutchinson , D. P. Wills , and P. Shipley . 2004. “MICRO‐CHECKER: Software for Identifying and Correcting Genotyping Errors in Microsatellite Data.” Molecular Ecology Notes 4: 535–538. 10.1111/j.1471-8286.2004.00684.x.

[ece374253-bib-0081] Wang, J. , W. Zhang , J. Wu , et al. 2022. “Multilocus Phylogeography and Population Genetic Analyses of *Opsariichthys hainanensis* Reveal Pleistocene Isolation Followed by High Gene Flow Around the Gulf of Tonkin.” Genes 13: 1908. 10.3390/genes13101908. 36292792 PMC9602001

[ece374253-bib-0082] Ward, R. D. , M. Woodwark , and D. O. F. Skibinski . 1994. “A Comparison of Genetic Diversity Levels in Marine, Freshwater, and Anadromous Fishes.” Journal of Fish Biology 44: 213–232. 10.1111/j.1095-8649.1994.tb01200.x.

[ece374253-bib-0083] Ward, R. D. , T. S. Zemlak , B. H. Innes , P. R. Last , and P. D. Hebert . 2005. “DNA Barcoding Australia's Fish Species.” Philosophical Transactions of the Royal Society, B: Biological Sciences 360: 1847–1857. 10.1098/rstb.2005.1716. PMC1609232 16214743

[ece374253-bib-0084] Webb, P. W. 1978. “Fast‐Start Performance and Body Form in Seven Species of Teleost Fish.” Journal of Experimental Biology 74: 211–226. 10.1242/jeb.74.1.211.

[ece374253-bib-0085] Wood, C. M. , and D. G. McDonald , eds. 1997. Global Warming: Implications for Freshwater and Marine Fish (No. 61). Cambridge University Press.

[ece374253-bib-0090] WWF . 2018. Living Planet Report 2018: Aiming Higher, edited by M. Grooten and R. E. A. Almond . WWF.

[ece374253-bib-0086] Yamanoue, Y. , D. H. E. Setiamarga , and K. Matsuura . 2010. “Pelvic Fins in Teleosts: Structure, Function and Evolution.” Journal of Fish Biology 77: 1173–1208. 10.1111/j.1095-8649.2010.02674.x. 21039499

[ece374253-bib-0087] Yoon, J. D. , J. H. Kim , S. H. Park , and M. H. Jang . 2018. “The Distribution and Diversity of Freshwater Fishes in Korean Peninsula.” Korean Journal of Ecology and Environment 51: 71–85. 10.11614/KSL.2018.51.1.071.

[ece374253-bib-0088] Zheng, Y. , B. Pan , X. Liu , et al. 2026. “Predicted Habitat Shifts and Conservation Priorities for Climate‐Sensitive Fish in Mountain Rivers Across a Climatic Transition Zone Under Future Climate Change.” Global Ecology and Conservation 67: e04132. 10.1016/j.gecco.2026.e04132.

