# Supplementary material for: Genetic (Genomic) and Morphological Evidence Suggest That the Korean Endemic Fat Minnow (Rhynchocypris kumgangensis) and the Deogyu Population Represent Distinct Species
Source: Ecol Evol. 2026 Sep 1;16(9):e74253. doi: 10.1002/ece3.74253 (PMC13534010; doi:10.1002/ece3.74253)
Supplement: Supplementary file 1 [file ECE3-16-e74253-s003.docx]

**Supplementary Table S1.** Information of sampling localities, river basins, location codes and latitude/longitude in this study.

|  | River basin | Location code | Location | Latitude | Longitude |
| --- | --- | --- | --- | --- | --- |
| *R. kumgangensis* | Han River | IB | Inje Bukcheon | 38°13'06.87"N | 128°22'15.30"E |
|  |  | PB | Pyeongchang Biancheon Stream | 37°42'10.93"N | 128°36'05.77"E |
|  |  | DN | Nam-cheon | 37°02'19.48"N | 128°30'46.47"E |
|  |  | YOD | Yeongwol Okdongcheon | 37°07'33.03"N | 128°50'03.57"E |
|  |  | HG | Hoengseong Gangrimcheon | 37°15'04.23"N | 128°04'58.20"E |
|  |  | JY | Jeongseon Yongtancheon | 37°25'36.64"N | 128°33'48.47"E |
|  |  | PG | Pyeongchang Gihwacheon | 37°17'29.46"N | 128°32'34.40"E |
|  |  | PH | Pyeongchang Heungjeongcheon | 37°39'23.67"N | 128°19'56.68"E |
|  |  | IBT | Inje Bangtaecheon | 38°00'55.55"N | 128°28'33.11"E |
|  | East-flowing  rivers | YO | Yangyang Osaekcheon | 38°04'36.86"N | 128°27'10.91"E |
|  |  | GY | Gangneung Yeongokcheon | 37°50'53.31"N | 128°43'27.48"E |
|  |  | UG | Ulsan Guksucheon | 35°35'41.09"N | 129°14'0.44"E |
| *R. deogyuensis* | Geum River | MG | Muju Gucheondong Valley | 35°52'47.88"N | 127°46'53.71"E |
